# Supplementary material for: Gender differences in the intention to study math increase with math performance
Source: Nat Commun. 2023 Jun 27;14:3664. doi: 10.1038/s41467-023-39079-z (PMC10300152; doi:10.1038/s41467-023-39079-z)
Supplement: Supplementary file 1 — Reporting Summary [file 41467_2023_39079_MOESM1_ESM.pdf]

nature portfolio

Corresponding author(s): Thomas Breda

Last updated by author(s): Oct 10, 2022

Reporting Summary

Nature Portfolio wishes to improve the reproducibility of the work that we publish. This form provides structure for consistency and transparency in reporting. For further information on Nature Portfolio policies, see our Editorial Policies and the Editorial Policy Checklist.

Statistics

For all statistical analyses, confirm that the following items are present in the figure legend, table legend, main text, or Methods section.

n/a	Confirmed	
☐	☒	The exact sample size (n) for each experimental group/condition, given as a discrete number and unit of measurement
☐	☒	A statement on whether measurements were taken from distinct samples or whether the same sample was measured repeatedly
☐	☒	The statistical test(s) used AND whether they are one- or two-sided<br>*Only common tests should be described solely by name; describe more complex techniques in the Methods section.*
☐	☒	A description of all covariates tested
☒	☐	A description of any assumptions or corrections, such as tests of normality and adjustment for multiple comparisons
☐	☒	A full description of the statistical parameters including central tendency (e.g. means) or other basic estimates (e.g. regression coefficient) AND variation (e.g. standard deviation) or associated estimates of uncertainty (e.g. confidence intervals)
☐	☒	For null hypothesis testing, the test statistic (e.g. F, t, r) with confidence intervals, effect sizes, degrees of freedom and P value noted<br>*Give P values as exact values whenever suitable.*
☒	☐	For Bayesian analysis, information on the choice of priors and Markov chain Monte Carlo settings
☒	☐	For hierarchical and complex designs, identification of the appropriate level for tests and full reporting of outcomes
☒	☐	Estimates of effect sizes (e.g. Cohen's d, Pearson's r), indicating how they were calculated

Our web collection on statistics for biologists contains articles on many of the points above.

Software and code

Policy information about availability of computer code

Data collection	The paper involves no data collection
Data analysis	The analysis was performed with standard statistical software. All the codes are ours and have been deposited at Breda, T., Jouini, E., & Napp, C. (2022). Replication package for "The gender gap in intentions to study math increases with math performance". Zenodo. DOI: https://doi.org/10.5281/zenodo.7181225

For manuscripts utilizing custom algorithms or software that are central to the research but not yet described in published literature, software must be made available to editors and reviewers. We strongly encourage code deposition in a community repository (e.g. GitHub). See the Nature Portfolio guidelines for submitting code & software for further information.

Data

Policy information about availability of data

All manuscripts must include a data availability statement. This statement should provide the following information, where applicable:

- Accession codes, unique identifiers, or web links for publicly available datasets
- A description of any restrictions on data availability
- For clinical datasets or third party data, please ensure that the statement adheres to our policy

The main data sources used in this research come from the 2012 Programme for International Student Assessment (PISA2012, https://www.oecd.org/pisa/pisaproducts/pisa2012database-downloadabledata.htm) and the Programme for the International Assessment of Adult Competencies (PIAAC, https://www.oecd.org/skills/piaac/publicdataandanalysis/). Both are managed by the OECD, and the data are publicly available. HSLS:09 data are publicly available at: https://nces.ed.gov/surveys/hsls09/. The robustness checks for France presented in Supplementary Table 9 are based on data whose access is restricted. To access these data, a request must sent to the statistical institute of the French Ministry of Education (the DEEP).

Field-specific reporting

Please select the one below that is the best fit for your research. If you are not sure, read the appropriate sections before making your selection.

☐ Life sciences ☒ Behavioural & social sciences ☐ Ecological, evolutionary & environmental sciences

For a reference copy of the document with all sections, see nature.com/documents/nr-reporting-summary-flat.pdf

Behavioural & social sciences study design

All studies must disclose on these points even when the disclosure is negative.

Study description	Observational study based on a large multi-country survey of more than 300,00 15 to 16 y.o. students in 61 countries. The survey (PISA) is conducted by the OECD. Additional analyses are done using a large multi-country survey of adult competencies (PIAAC) and country-specific data on student enrolment.
Research sample	The main research sample includes 251,120 15 to 16 y.o. female and male students in 61 countries. It is representative of the student population in the studied countries (using provided sampling weights). It allows us to study how educational intentions vary by gender in a large set of countries at the age when important educational decisions are taken. Auxiliary research samples include working adults and high-school students in France.
Sampling strategy	The sampling for PISA is stratified by school. Participating schools in each country are first selected. Then, the predetermined target cluster size in each school was 42 students for most schools that did a computer-based assessment, and 35 for most schools that did a paper-based assessment. Sampling weights are calculated accordingly. Further details can be found at https://www.oecd.org/pisa/pisaproducts/pisa2012technicalreport.htm
Data collection	Data collection in PISA was both paper-based and computer-based. It was done by the OECD which has a long experience in running the PISA survey. This survey was launched in 2000 and occurs every three years. All details about data collection are provided in the PISA technical report: https://www.oecd.org/pisa/pisaproducts/pisa2012technicalreport.htm. PISA was not initially intended to test the hypotheses we examine in this paper, implying that data collection could not be influenced by these hypotheses.
Timing	Data collection occured during the year 2012 from 1 March 2012 to 31 December 2012
Data exclusions	In our main analyses, we only exclude students that did not provide their intentions to pursue math-related studies or careers.
Non-participation	The school response rate target was 85 percent for all education systems. There was a target of 85 percent of schools participating from the original sample of schools. PISA 2012 also required a minimum participation rate of 80 percent of sampled students from schools within each education system, implying that participation is at least 80%. Non-participation is primarily due to sickness absences (the student does not show up the day of the test or questionnaire). See details at https://www.oecd.org/pisa/pisaproducts/pisa2012technicalreport.htm
Randomization	Our study is a large observational study providing non-causal relationships. It does not involve randomization of any kind.

Reporting for specific materials, systems and methods

We require information from authors about some types of materials, experimental systems and methods used in many studies. Here, indicate whether each material, system or method listed is relevant to your study. If you are not sure if a list item applies to your research, read the appropriate section before selecting a response.

Materials & experimental systems

n/a	Involved in the study	
☒	☐	Antibodies
☒	☐	Eukaryotic cell lines
☒	☐	Palaeontology and archaeology
☒	☐	Animals and other organisms
☒	☐	Human research participants
☒	☐	Clinical data
☒	☐	Dual use research of concern

Methods

n/a	Involved in the study	
☒	☐	ChIP-seq
☒	☐	Flow cytometry
☒	☐	MRI-based neuroimaging
