# Supplementary material for: Gender differences in the intention to study math increase with math performance
Source: Nat Commun. 2023 Jun 27;14:3664. doi: 10.1038/s41467-023-39079-z (PMC10300152; doi:10.1038/s41467-023-39079-z)
Supplement: Supplementary file 2 — Supplementary Information [file 41467_2023_39079_MOESM2_ESM.pdf]

Supplementary Information for

Gender differences in the intention to study math increase with math performance

Authors: Thomas Breda[1,2], Elyès Jouini[3,2], Clotilde Napp[2,3]

Affiliations:

[1] Paris School of Economics

[2] CNRS

[3] Université Paris Dauphine

Table of Content

Supplementary Note 2

Theoretical properties of boys' and girls' pattern of intentions ensuring a negative impact on girls' relative performance and representation among those who intend to do math 2

Empirical confirmation of the negative impact of the differential pattern of intentions alone on girls' relative performance and representation at high levels 3

High School Longitudinal Study and actual enrolment of high school students 4

Supplementary Figures 7

Supplementary Tables 21

Supplementary Note

First, we verify that differential intentions by themselves lead to girls' lower math performance and lower representation among the highly able, within the population of those (boys and girls) who intend to do math. For this purpose, we start by exhibiting a theoretical condition on girls and boys intentions to do math that ensures a gender gap in performance and a lower representation of girls among top performers, within the population of those (boys and girls) who intend to do math, whereas this is not the case in the overall population. Then, we confirm empirically the negative effect of differential intentions alone on girls' relative performance

Second, we exploit a dataset from the High School Longitudinal Study (HSLS:09), to shed light on the validity of our conclusions on actual enrolment.

Theoretical properties of boys' and girls' pattern of intentions ensuring a negative impact on girls' relative performance and representation among those who intend to do math

We let Condition C denote the fact that the ratio between boys' and girls' conditional probabilities to intend to do math given their math performance is an increasing function of this performance.

Condition C corresponds to the fact that the gender gap in intentions increases with math performance, when the gender gap is measured by the ratio of boys' and girls' probability of strongly intending to do math. We have seen in the main text that this condition is satisfied by our intentions data (see Figure 2).

Proposition *If boys' and girls' probabilities to intend to do math satisfy condition C and if boys and girls have the same initial distribution of performance, then*

1. *the average math performance of boys among those who intend to do math is higher than that of girls;*
2. *among those intending to do math, the ratio of girls to boys above a given level of performance decreases with that level.*

Proof By Condition C, $\frac{P_b[(\mathrm{C}=1)|(Y=x)]}{P_g[(C=1)|(Y=x)]}$ is an increasing function of x, where $C \in \{0,1\}$ denotes the intention to do math, Y denotes math performance, and P_b and P_g respectively denote the probability measures for boys and girls.

We have $P_g[(Y=x)|(C=1)] = \frac{P_g[(Y=x)\cap(C=1)]}{P_g[(C=1)]} = \frac{P_g[(C=1)|(Y=x)]P_g[(Y=x)]}{P_g[(C=1)]}$ as well as the analogous relation for boys. Assuming the same ability distribution for boys and girls, we have $P_g[(Y=x)] = P_b[(Y=x)]$, hence the equivalence between Condition C and the fact that the function $\frac{P_b[(\mathrm{Y}=x)|(C=1)]}{P_g[(Y=x)|(C=1)]}$ is nondecreasing in x, or the MLR-dominance of the distribution of boys' math performance over that of girls among those intending to do math.

1. The average performance A_b of boys among those intending to do math is given by $A_b = E_b[\mathrm{Y}|\mathrm{C}=1]$. We can write A_b in the form $A_b = E_G[Yh(Y)]$, for a function h such that $h(x) = \frac{P_b[(\mathrm{Y}=x)|(C=1)]}{P_g[(Y=x)|(C=1)]}$ and a probability measure P_G such that $P_G(Y=\mathrm{x}) = P_g[(Y=x)/(C=1)]$. We know that for two random variables X and Z such that Z can

be written in the form of an increasing function of X, we have $E[XZ] \geq E[X]E[Z]$, where the expected value is taken under any probability measure. Under Condition C, h is increasing, hence $A_b = E_G[Yh(Y)] \geq E_G[Y]E_G[h(Y)]$. Now, $E_G[Y] = E_g[\mathrm{Y|C} = 1] = A_g$, where we let A_g denote the average performance of girls among those intending to do math, and $E_G[h(Y)] = 1$. This means that $A_b \geq A_g$ and the first assertion holds.

2. The ratio R_x of girls to boys among those intending to do math above a given level of performance x is given by $R_x = \frac{g \times \sum_{y \geq x} P_g[(Y=x)|(C=1)]}{b \times \sum_{y \geq x} P_b[(Y=x)|(C=1)]}$, where g and b denote the number of girls and boys intending to do math. The behavior of R_x as a function of x is the same as that of $\frac{S_x^g}{S_x^b} = \frac{\sum_{y \geq x} P_g[(Y=x)|(C=1)]}{\sum_{y \geq x} P_b[(Y=x)|(C=1)]}$. Let x be a given performance level and $x + h$ the performance level that is immediately above. Direct computations give that $\frac{S_x^g}{S_x^b} - \frac{S_{x+h}^g}{S_{x+h}^b}$ has the same sign as $\frac{P_g[(Y=x)|(C=1)]}{P_b[(Y=x)|(C=1)]} - \frac{S_{x+h}^g}{S_{x+h}^b}$. Now, $\frac{S_{x+h}^g}{S_{x+h}^b}$ can be considered as a weighted average of the $\frac{P_g[(Y=y)|(C=1)]}{P_b[(Y=y)|(C=1)]}$ for $y > x$, where the weights are proportional to $P_b[(Y = y)|(C = 1)]$. Since the function $\frac{P_g[(\mathrm{Y}=x)|(C=1)]}{P_b[(\mathrm{Y}=x)|(C=1)]}$ is decreasing in x, we get that $\frac{P_g[(Y=x)|(C=1)]}{P_b[(Y=x)|(C=1)]} \leq \frac{S_{x+h}^g}{S_{x+h}^b}$, hence $\frac{S_x^g}{S_x^b} - \frac{S_{x+h}^g}{S_{x+h}^b}$ is positive and the ratio R_x of girls to boys among those intending to do math above a given level of performance x decreases with that level. ■

Note that the proof is made considering discrete distributions. Straightforward adaptations permit to derive the same results with continuous distributions. Similarly, the same results can be obtained replacing the performance by the fractile of performance. Indeed, the fractile of performance is just a nondecreasing (increasing) transformation of the performance (when the subdivision in fractiles is thin enough, i.e. when the number of fractiles is large enough).

Empirical confirmation of the negative impact of the differential pattern of intentions alone on girls' relative performance and representation at high levels

As explained in the main text, in order to pin down the impact of the differential pattern of intentions alone, we need to net out the impact of the differences in ability distribution. We do so in panel B of Supplementary Table 7 that provides statistics after reweighting students so that girls and boys have the same ability distributions. The reweighting is done separately for each plausible value (PV) of math ability, so that we construct five sets of weights (one for each PV), compute the relevant statistics for each plausible value and its associated weight, and average the results over all PVs. For each PV, we reweight girls with a standard DFL reweighting procedure so that their ability distribution becomes similar to that of boys. In practice, we do so by applying a weighted linear regression (using PISA students' weights W) of a dummy for girls on one hundred dummies for each percentile of the ability distribution (for the relevant PV). We then take the predicted value p(girl). The final weight is W for boys and W*((1-p(girl))/p(girl))* ((1-s) / s) for girls where s is the share of girls in the sample (weighting observations by W). This whole weighting procedure is applied separately on each sample on which we provide statistics (all countries, OECD countries and non-OECD countries).

Therefore, these statistics cannot capture differences in ability distributions. Panel A of Supplementary Table 7 provides similar statistics without reweighting to allow for easy comparisons, as in Table 2. Column (a) shows a gender gap in performance, at the advantage of boys, before selection occurs in the general population (panel A), but not anymore with reweighting making boys' and girls' ability distributions alike (panel B). Comparing columns (d) and (f) of Supplementary Table 7, one can see that girls-to-boys ratios in the general population are decreasing with math performance (panel A), but not anymore once distributions of math ability have been equated (panel B).

The comparison of columns (a) and (b) shows the negative impact of intentions on girls' relative math performance. In panel B, this result cannot be attributed to initial differences in abilities. We observe that the negative impact of the differential pattern of intentions itself is more marked on non-OECD countries. When we consider all PISA 2012 participating countries, the differential intentions alone (when initial distributions are identical) leads to gender performance gap among those strongly intending to pursue math studies of 3.7% of a standard deviation. This represents approximately 70% of the total increase in the gender gap in math performance observed after selection in panel A.

Columns (d) and (e) of Supplementary Table 7 then compare girls-to-boys ratios in the whole population and among those strongly intending to pursue math studies and show how the girls-to-boys ratio is strongly reduced among these due to the fact that intentions to pursue math studies are lower among girls (factor (ii)).

Finally, the comparison of columns (f) and (e) shows the impact of selection on girls' representation among high performers. The girls-to-boys ratio among those strongly intending to pursue math studies is decreasing with math ability. In panel B, this result cannot be attributed to initial differences in abilities. We see that the decrease remains limited, so the small girls-to-boys ratio among those strongly intending to pursue math studies above any level of math ability is primarily due to the fact that, on average, intentions to pursue math studies are lower among girls.

High School Longitudinal Study and actual enrolment of high school students

We also exploit a dataset from the High School Longitudinal Study (HSLS:09), a US nationally representative study of the National Center for Education Statistics (NCES) designed with a focus on STEM. Note that this dataset is analyzed in (19), restricting the sample to 4-year college students.

HSLS:09 is a longitudinal study of approximately 20,000 ninth graders. Students were first surveyed in fall 2009 as ninth graders and were surveyed again 2.5 years later, in 2012, when most were spring-term eleventh graders. In the summer and fall of 2013, students or their parents responded to a survey about the student’s high school completion status, postsecondary education and work plans, college application experiences, and work experiences. In addition, school personnel in base-year schools and other schools identified during data collection supplied high school transcripts for HSLS:09 students from all schools that these students had attended.

We consider as outcome the actual enrolment of high school students in a given set of courses in engineering, computer science and math, as declared in 2013. These outcomes are particularly important since the leaky pipeline starts at high school level and high school choices shape college choices (see e.g., 14). We observe for instance on HSLS data that a much higher percentage of males than females earn any high school credit in engineering and technology (21% versus 8%)

More precisely, we consider as outcome the earning of an above average number of credits in math (i.e., 3.5) as well as either more than one credit in computer science or at least one credit in engineering. We say that the student is in MECS (Math, Engineering or Computer Science) if he has earned these credits. We obtain similar results if we restrict our attention to engineering credits only or computer science credits only. We consider as a measure of math performance the score at a math assessment that students take during 9th grade ("theta math ability score", fall 2009). As a robustness check, we also consider the math score at the first follow up (11th grade, spring 2012). We use the longitudinal sampling weights provided by HSLS.

Our sample consists of 16,303 students (50% females), with available data for math scores and credits. On average, around 17% of males but only 9% of females are in MECS.

Supplementary Figure 6 presents the proportion of males, of females, and of all students in MECS as a function of the deciles of math performance. The features are very similar to those of Figure 1 in the main text. In particular, the relation is more increasing for males than for females. The probability of being in MECS varies from 0.07 to 0.1 for females and from 0.07 to 0.27 for males. Moreover, math performance explains better MECS for males than for females. Indeed, the R-squared of a simple regression of MECS on math performance is much larger for males than for females (0.03 vs. 0.001).

As a result of the differential enrolment of females and males into MECS, the difference between the proportion of males and the proportion of females in MECS increases with math performance, as is illustrated by Supplementary Figure 7. The sex gap is very limited and not statistically different from zero for the lowest levels of math ability but it increases steadily and linearly with math ability, from close to 0 for the lowest achievers to 15% for the highest achievers.

We fit linear models of the type:

$$MCES_i = \alpha Girl_i + \beta Math\_ability_i + \gamma(Math\_ability_i * Girl_i) + \varepsilon_i$$

(Supplementary Equation 1)

Supplementary Equation (1) captures how the probability of enrolling in MECS varies with math ability for males (coefficient β) and for females ($\beta + \gamma$), assuming that the relationship is linear for both sexes. A one standard deviation increase in math performance increases males' probability to enroll in MECS by 6 percentage points, but females' probability by only 1 percentage point.

Supplementary Figure 8 presents the ratio between the proportion of males and the proportion of females in MECS as a function of the deciles of math performance. We observe that this ratio is indeed increasing with math performance, being close to 1 among the poor performers and reaching 2.5 for high performers.

In terms of relative performance, in the whole population and among the students in MECS, we obtain the following result. The sex gap in math performance is null among all high schoolers but it amounts to 0.25SD at the advantage of males and is highly significant among those who choose these courses. If we consider as a measure of math performance the score at the second assessment (in 2012), the result is even stronger with a sex gap of 0.35SD.

These results mirror those we obtain in the main text about intentions to pursue math studies and careers. They suggest that our conclusions remain valid for actual course choices in high school in the US.

Supplementary Figures

Supplementary Figure 1: Gender gap in strong intentions to do math as a function of math performance. Selected countries/regions

a) OECD countries only

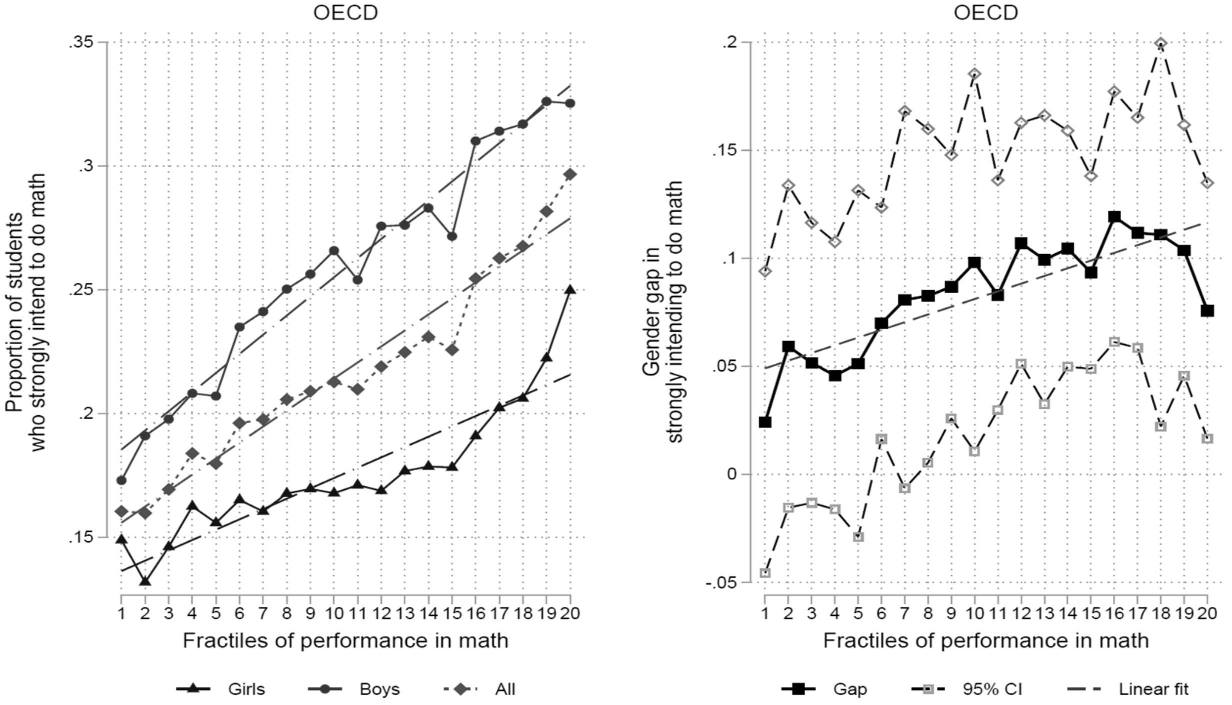

b) Non-OECD countries only

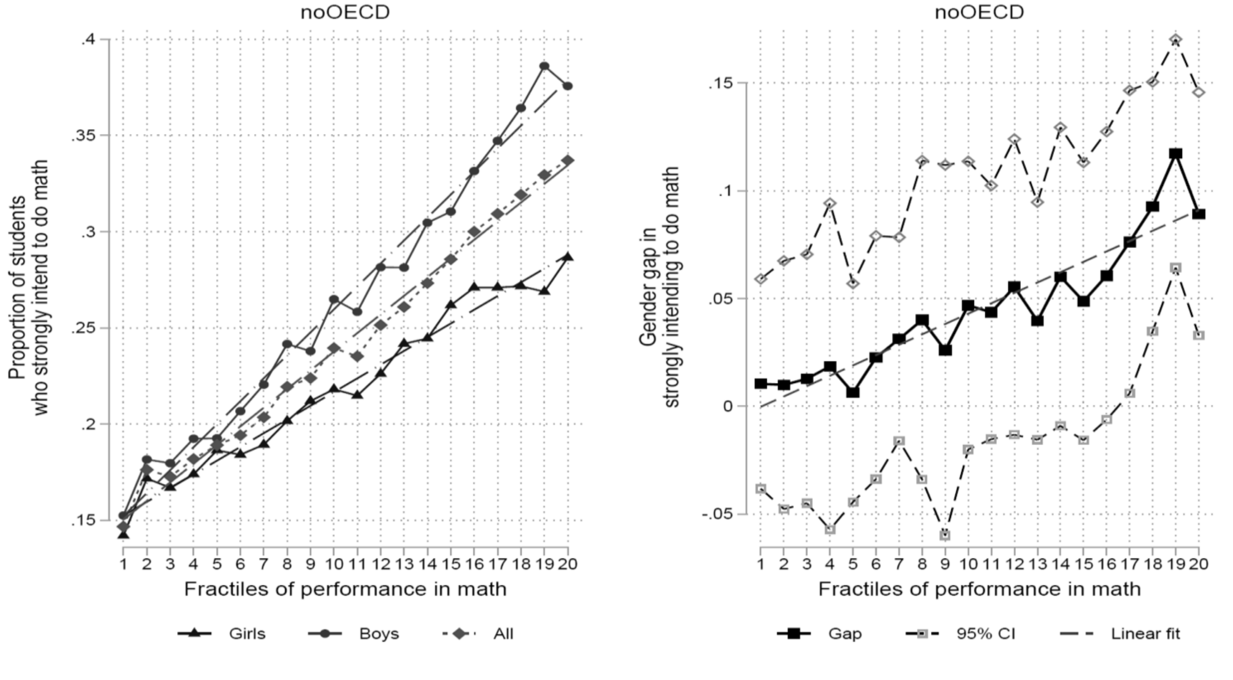

c) United-States

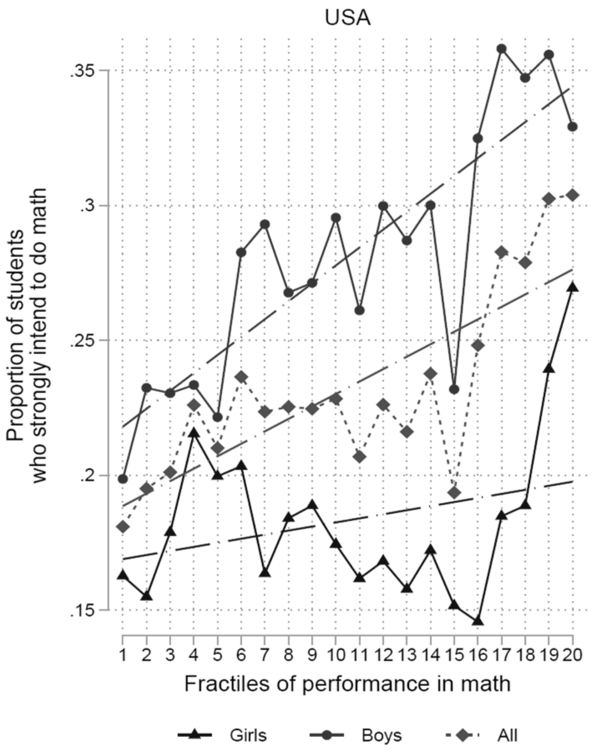

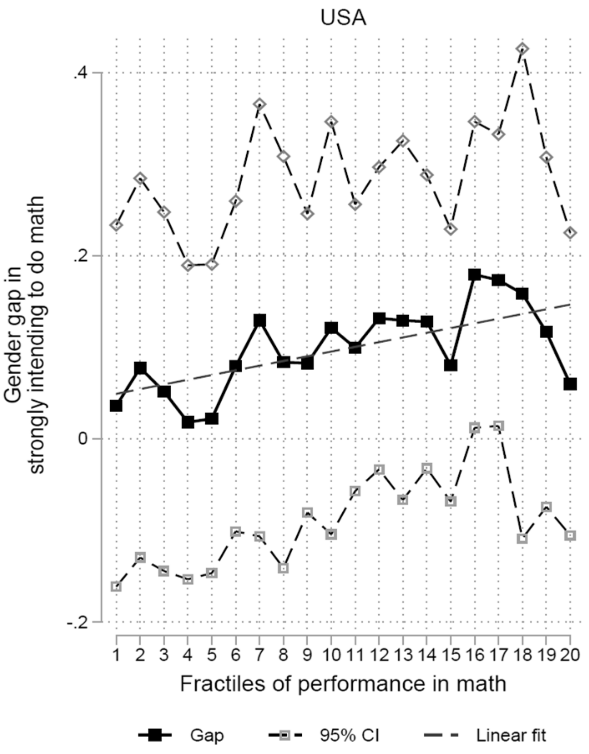

d) France

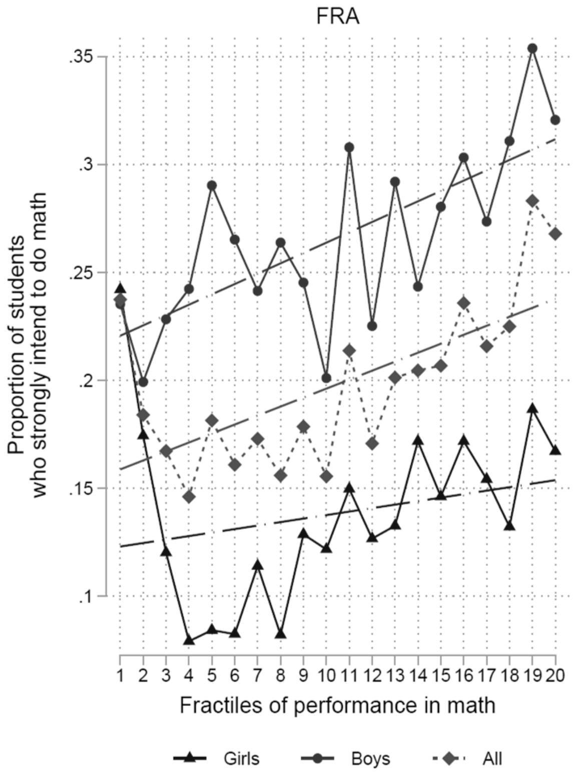

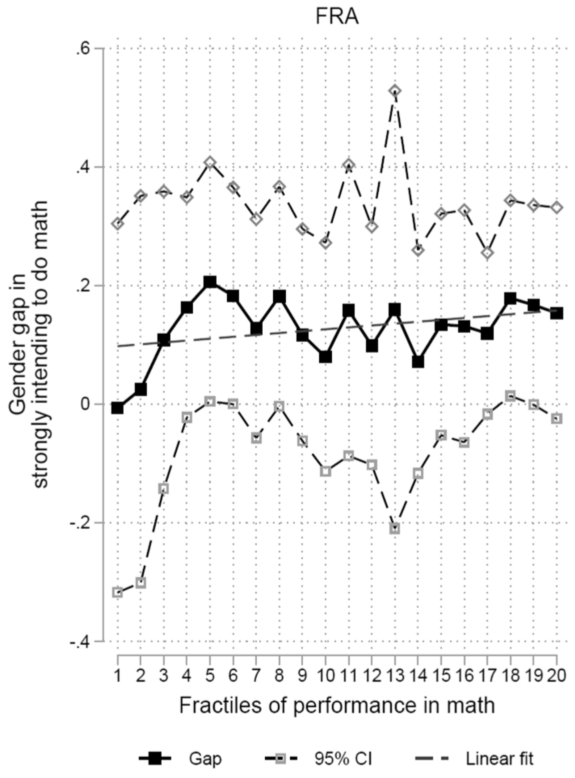

e) Brazil

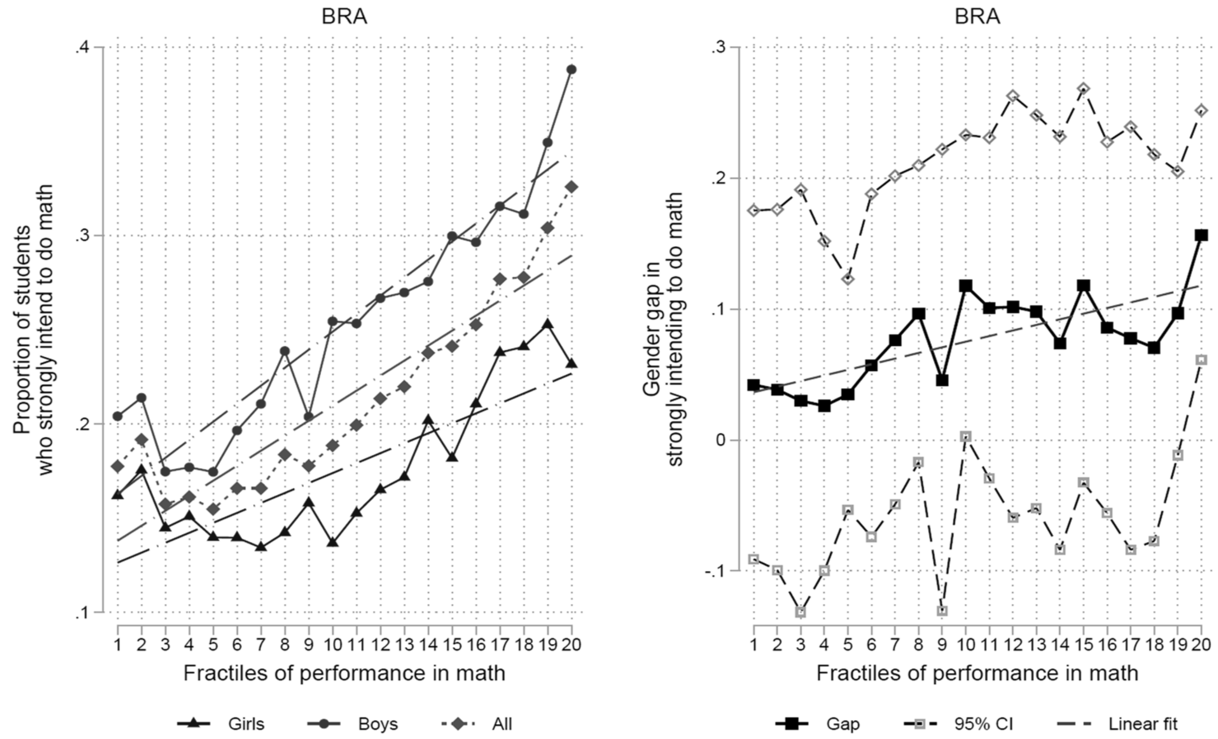

f) Russia

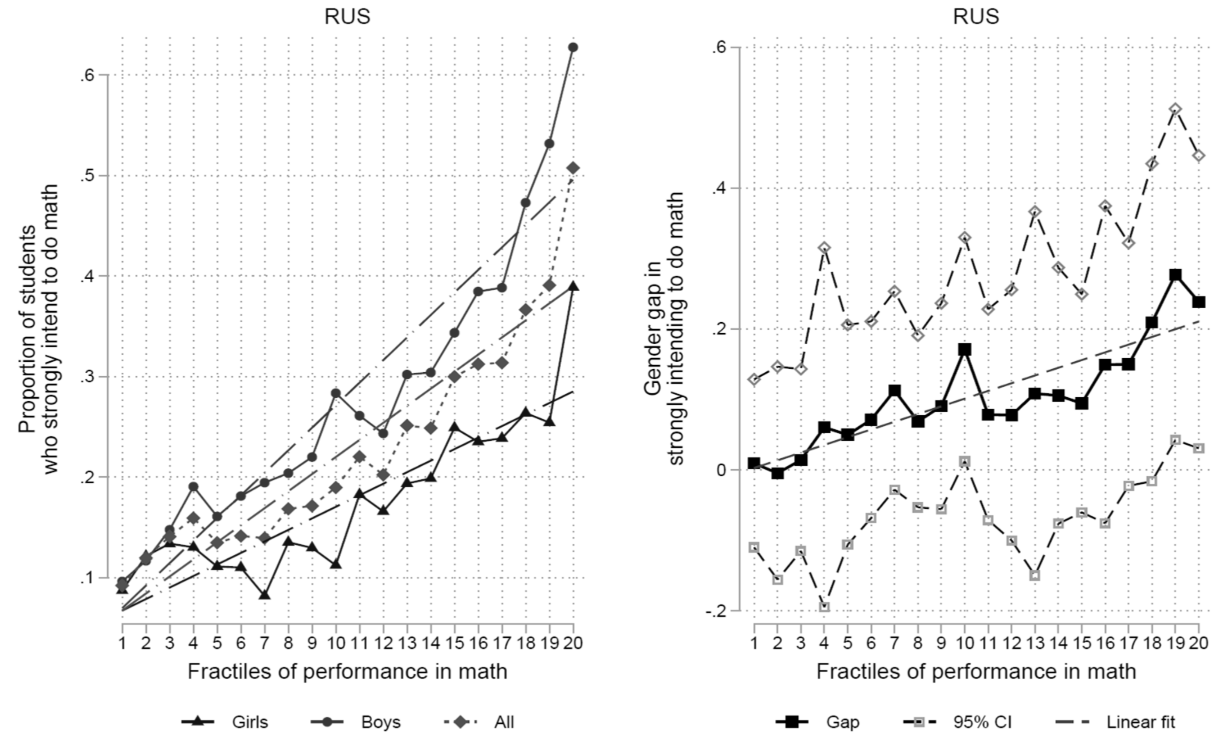

Figures a) to f) represent the same analysis as in Figure 1 (see the legend of Figure 1) for specific countries or groups of countries: a) OECD, b) Non-OECD, c) US, d) France, e) Brazil, f) Russia. Confidence intervals are based on two-sided t-tests.

Supplementary Figure 2: Girls' lower representation at high levels of math performance among students strongly intending to do math

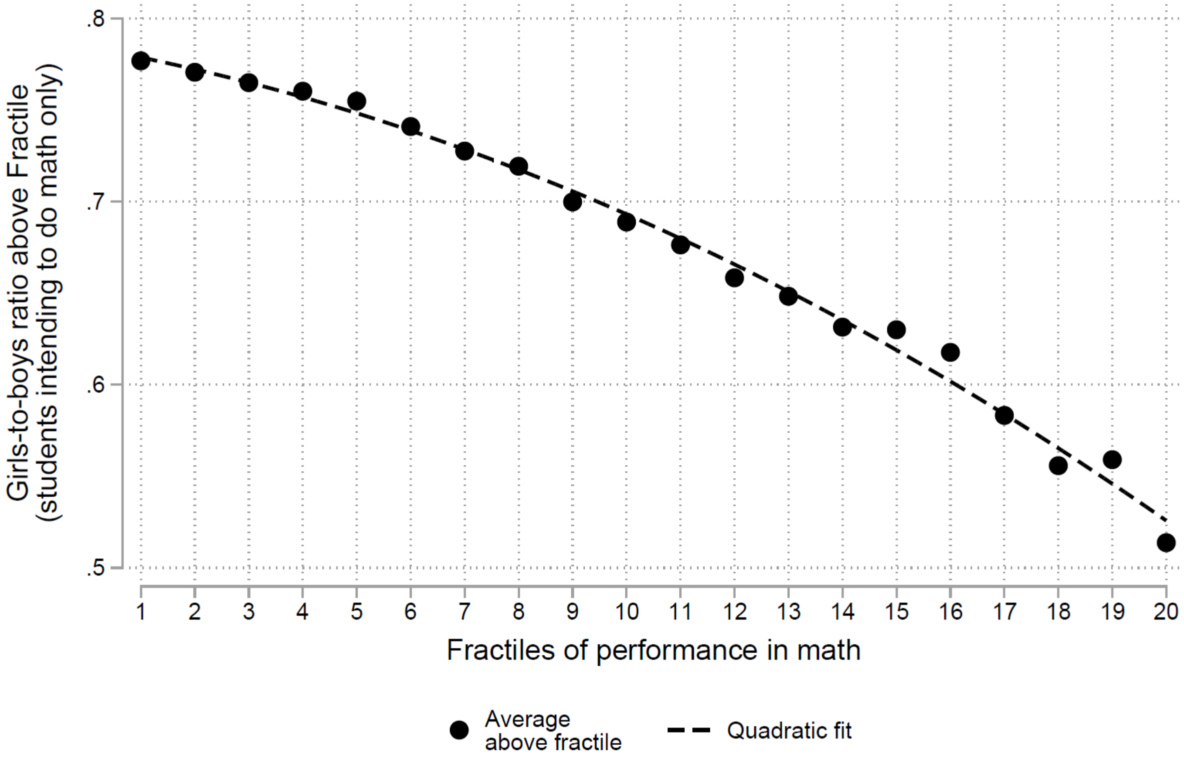

This figure represents the girls-to-boys ratio of students strongly intending to do math, above a given fractile of performance. See the legend of Figure 1 for more details about the fractiles of performance and the definition of "strongly intending to do math".

Supplementary Figure 3: Gender gap in intentions to do math as a function of math performance not standardized by country

(3a: Girls and boys separately) **(3b: Gender gap)**

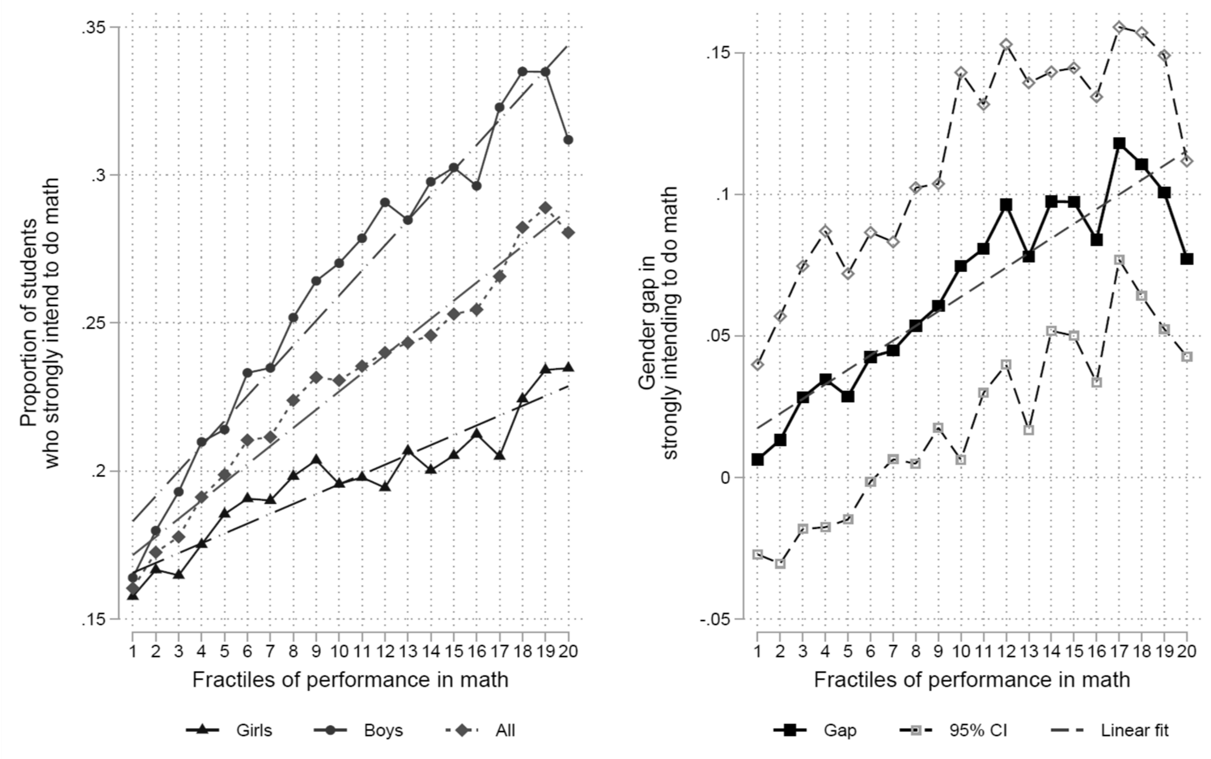

This figure is the analogue of Figure 1 when we consider a measure of math performance that is not standardized by country. Here, math performance is standardized to have a weighted mean equal to zero and a weighted standard deviation equal to one in the whole sample of 61 countries. See the legend of Figure 1 for other details. Confidence intervals are based on two-sided t-tests.

Supplementary Figure 4: Gender gap in intentions to do math as a function of math performance fractiles of students' gender-specific performance distributions.

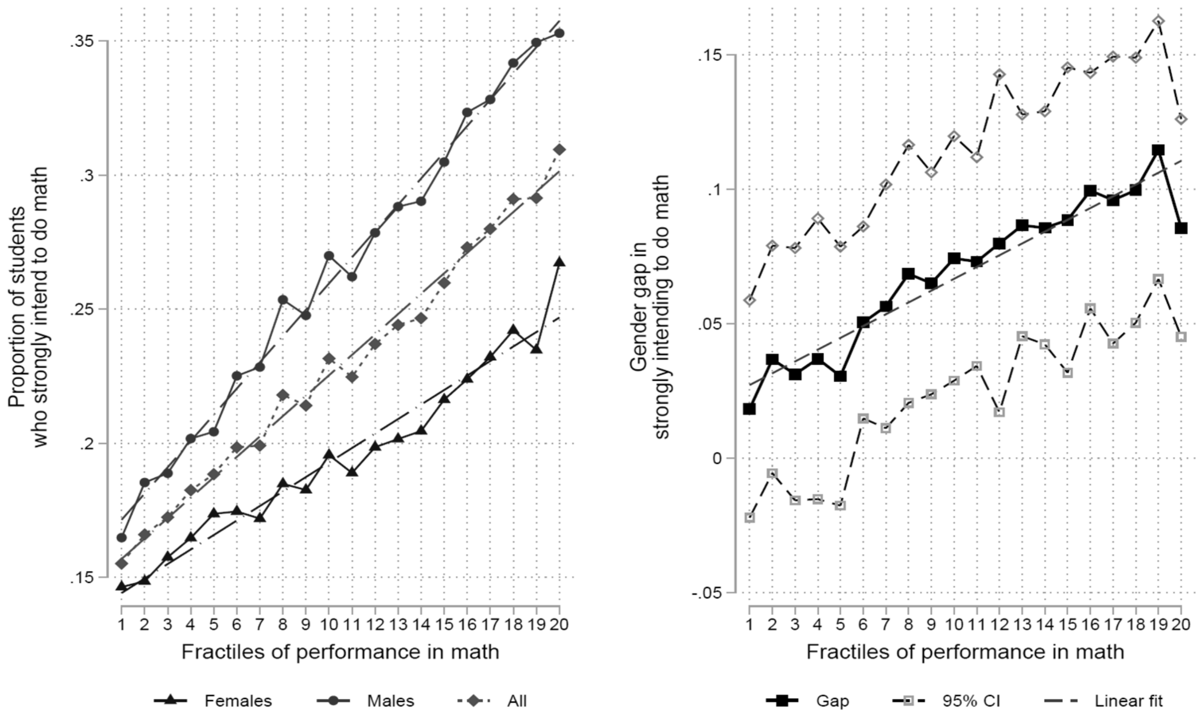

This figure is the analogue of Figure 1 except that the math performance fractiles considered in this figure are gender specific. The Figure therefore compares girls and boys that are at the same rank in their gender-specific math performance distribution rather than at the same rank in the whole math performance distribution. Confidence intervals are based on two-sided t-tests.

Supplementary Figure 5: Sex gap in enrollment in math-related fields as a function of math performance. Results for France based on math performance measured at the end of high school and actual enrollment in higher education.

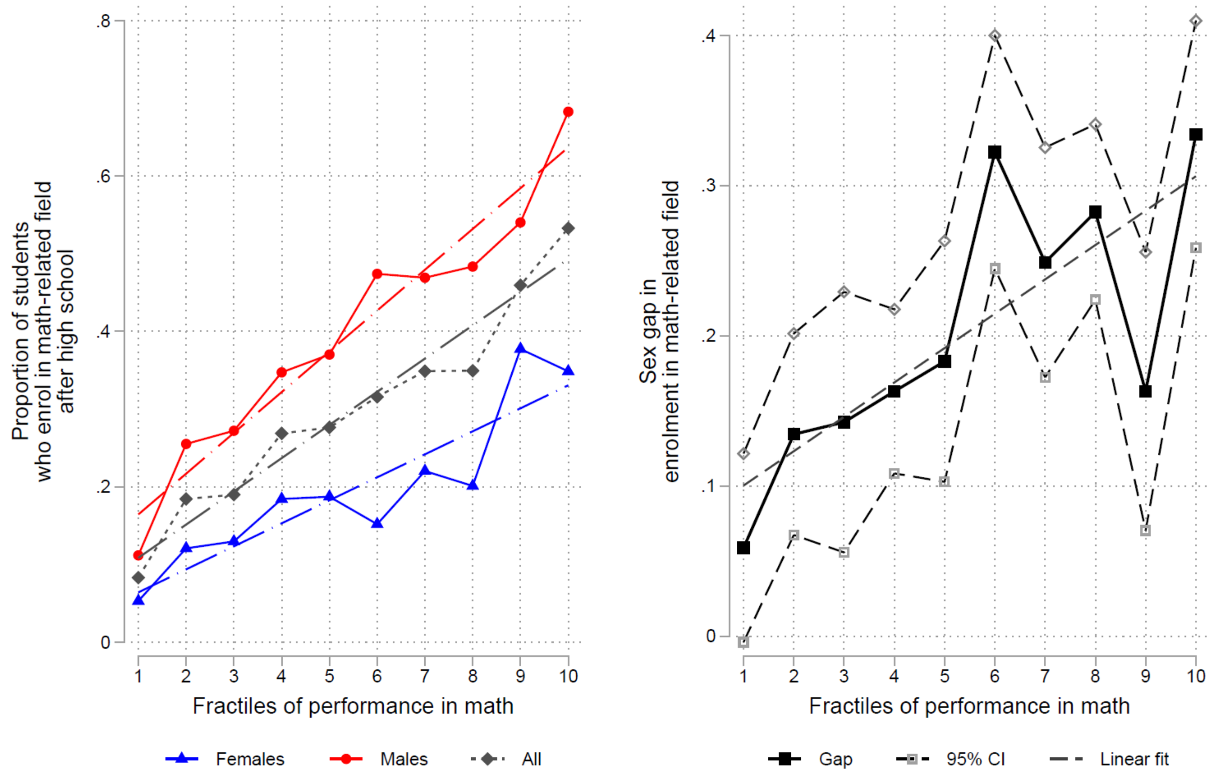

Performance in math is the students' math grade obtained at the *Baccalauréat* math test. Math-related fields correspond to university and selective higher education programs in mathematics, physics, engineering or computer sciences. The sample consists of 5,574 students (due to this smaller sample size we split it in 10 groups instead of 20 groups as we do in the main figures). Confidence intervals are based on two-sided t-tests.

Supplementary Figure 6: MECS (Math, Engineering and Computer Science in HSLS:09) for males and for females as a function of math performance

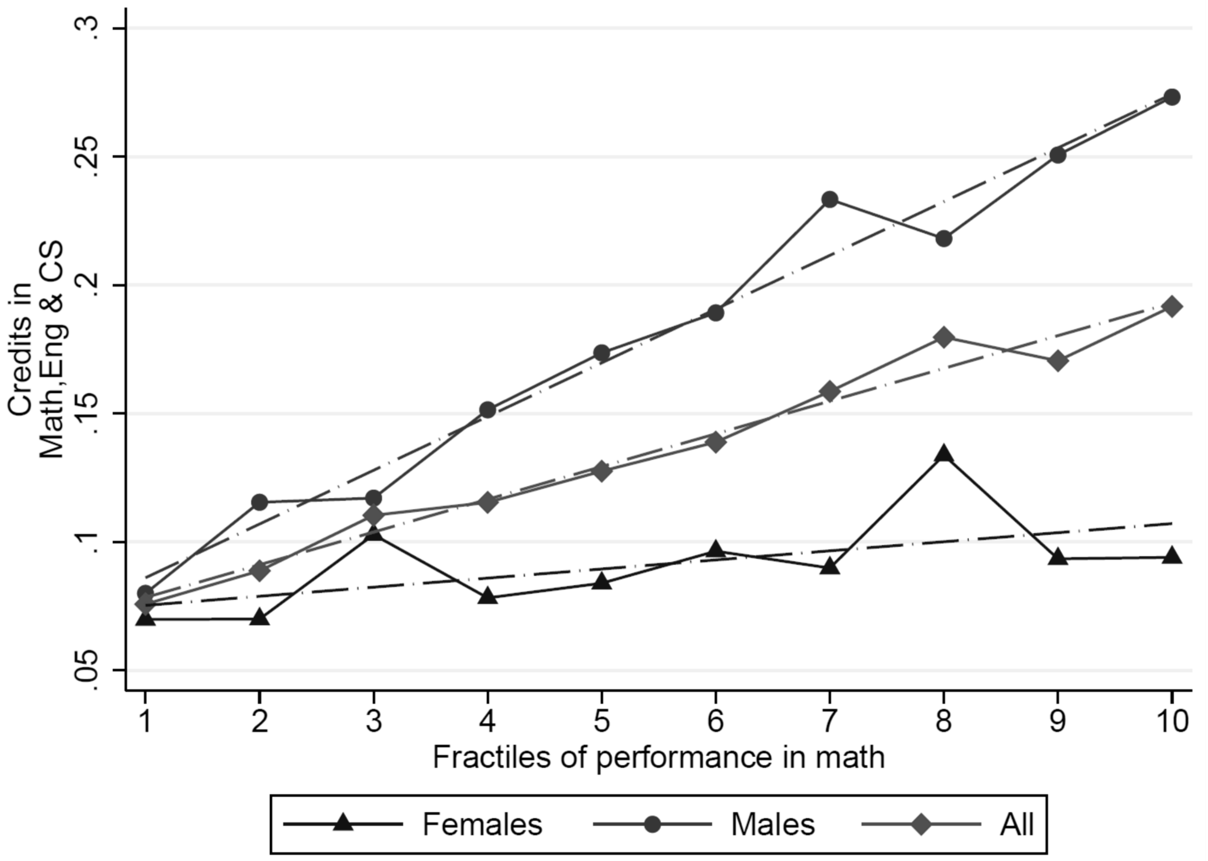

The sample consists of 16,303 students, Performance in math is the students' math score obtained at the 9th grade assessment. The Figure shows the percentage of males and of females earning above a given number of credits in high school in Math, Engineering or Computer Science by decile of math performance.

Supplementary Figure 7: Sex gap in MECS (Math, Engineering and Computer Science in HSLS:09) as a function of math performance

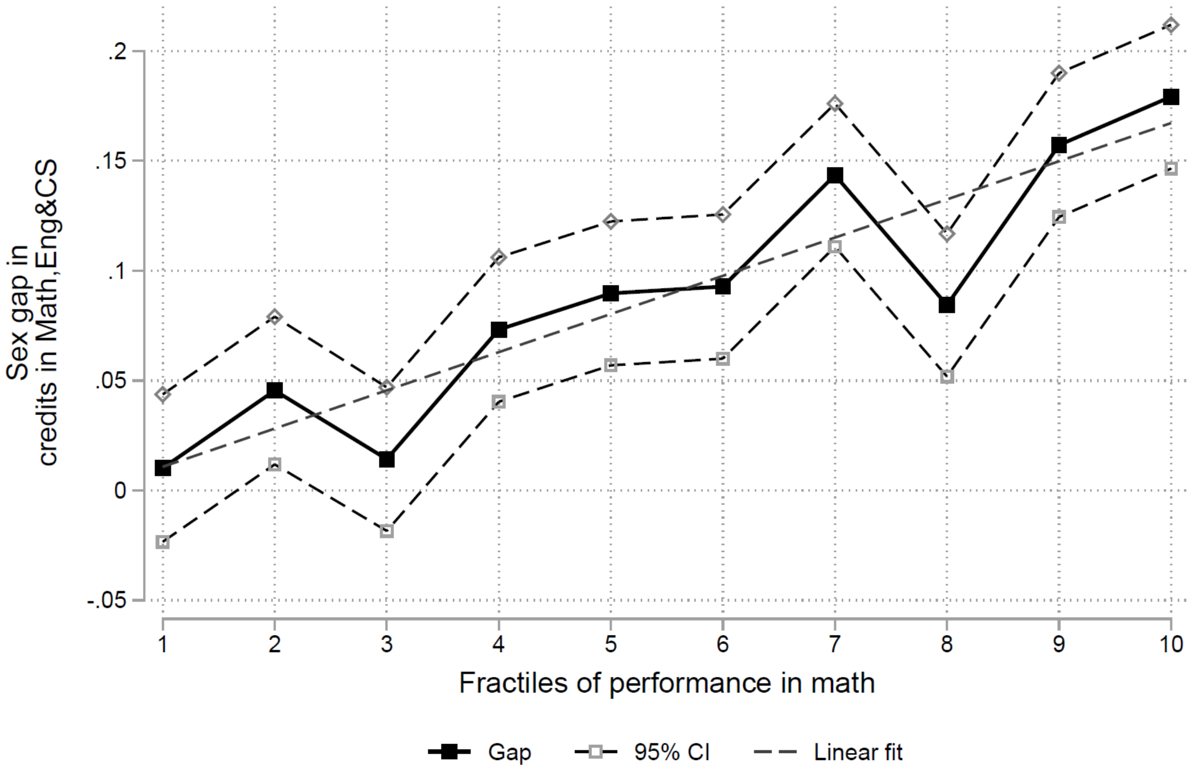

The sample consists of 16,303 students, Performance in math is the students' math score obtained at the 9th grade assessment. The Figure shows the difference between the percentage of males and the percentage of females earning above a given number of credits in high school in Math, Engineering or Computer Science by decile of math performance. Confidence intervals are based on two-sided t-tests.

Supplementary Figure 8: Males-to-females ratio in MECS (Math, Engineering and Computer Science in HSLS:09) as a function of math performance

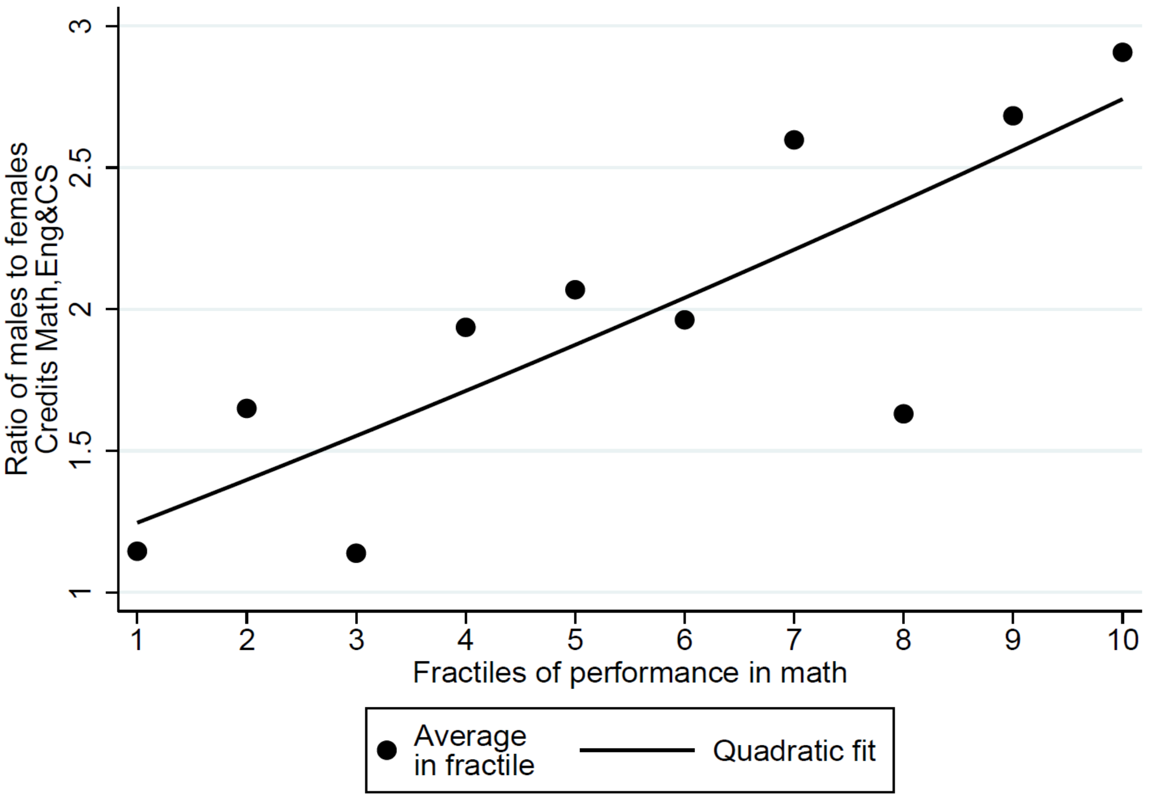

The sample consists of 16,303 students, Performance in math is the students' math score obtained at the 9th grade assessment. The Figure shows the ratio between the percentage of males and the percentage of females earning above a given number of credits in high school in Math, Engineering or Computer Science by decile of math performance.

Supplementary Figure 9a: Sex gap in the probability to work in a math-related occupation as a function of math performance

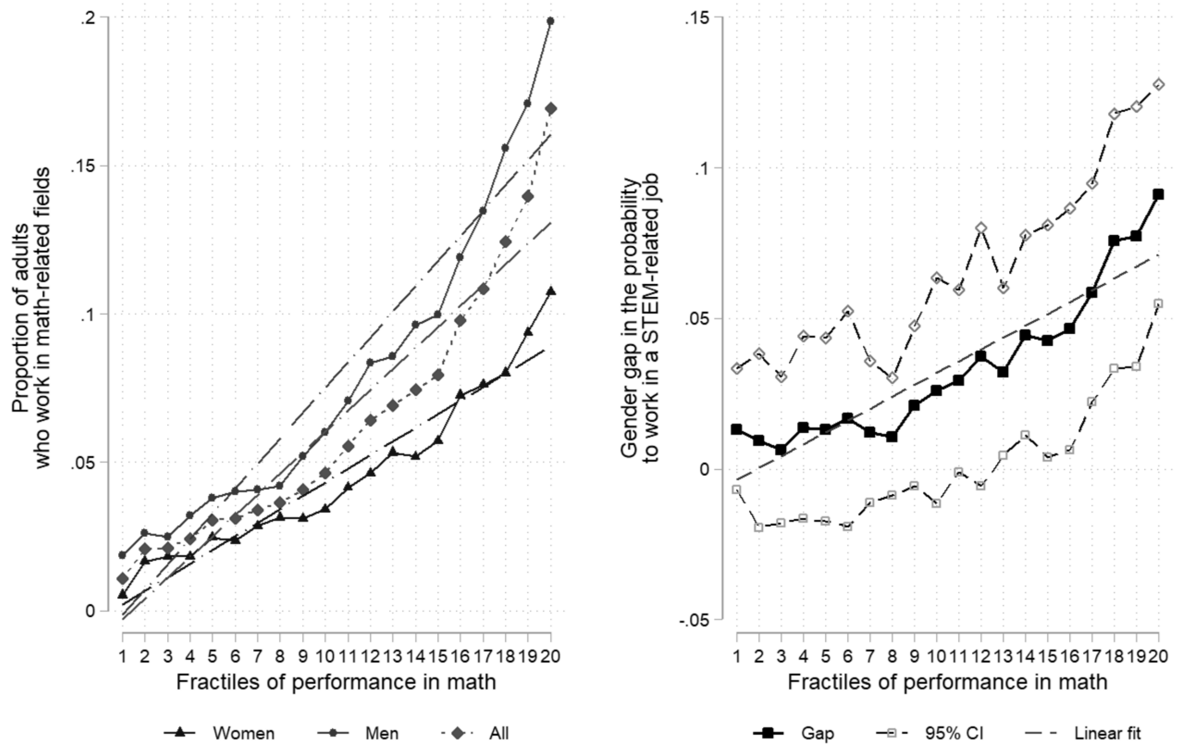

Supplementary Figure 9b: Sex gap in the probability to work in a *STEM* occupation as a function of math performance

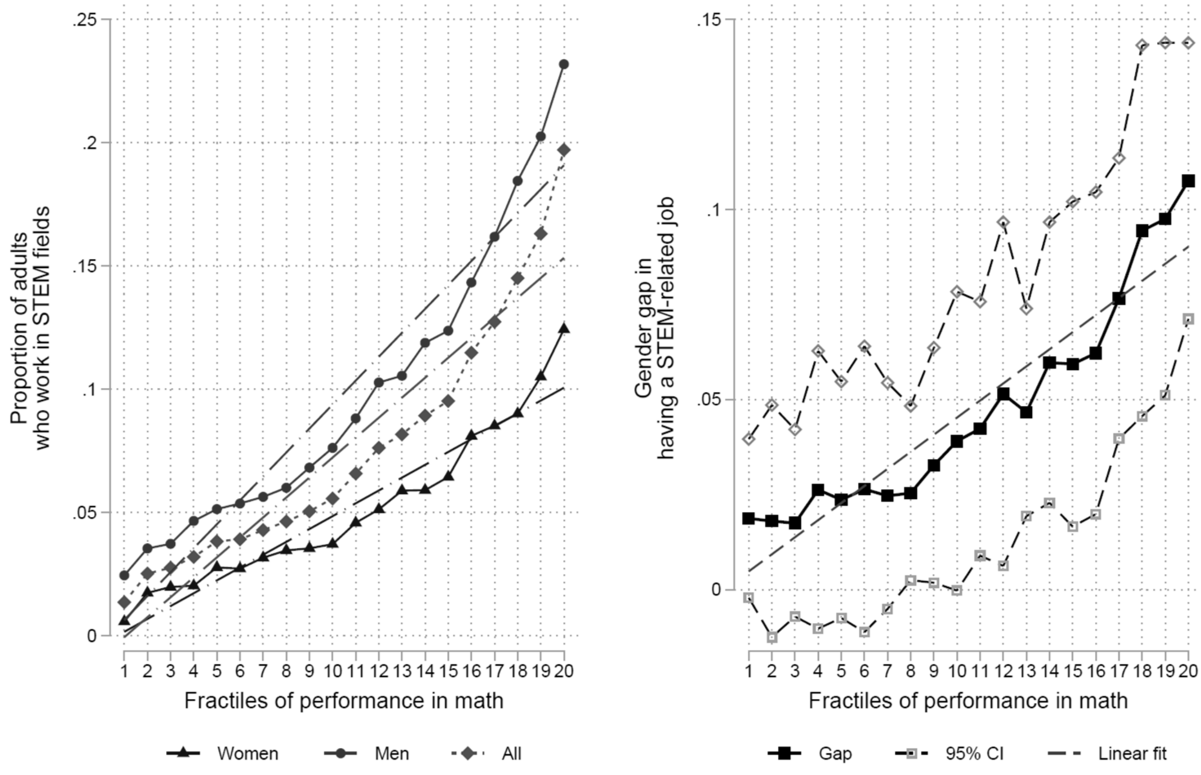

Supplementary Figure 9c: Sex gap in the probability to use numeracy skills intensively at work as a function of math performance

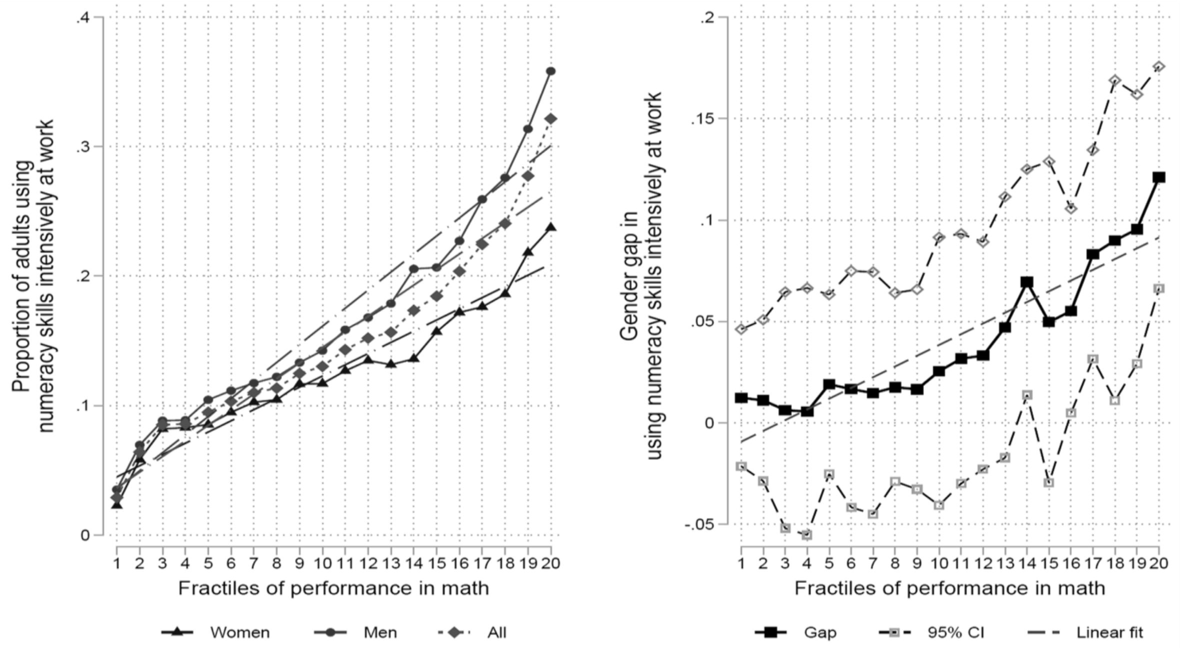

Supplementary Figure 9d: Sex gap in usage of numeracy skills at work as a function of math performance

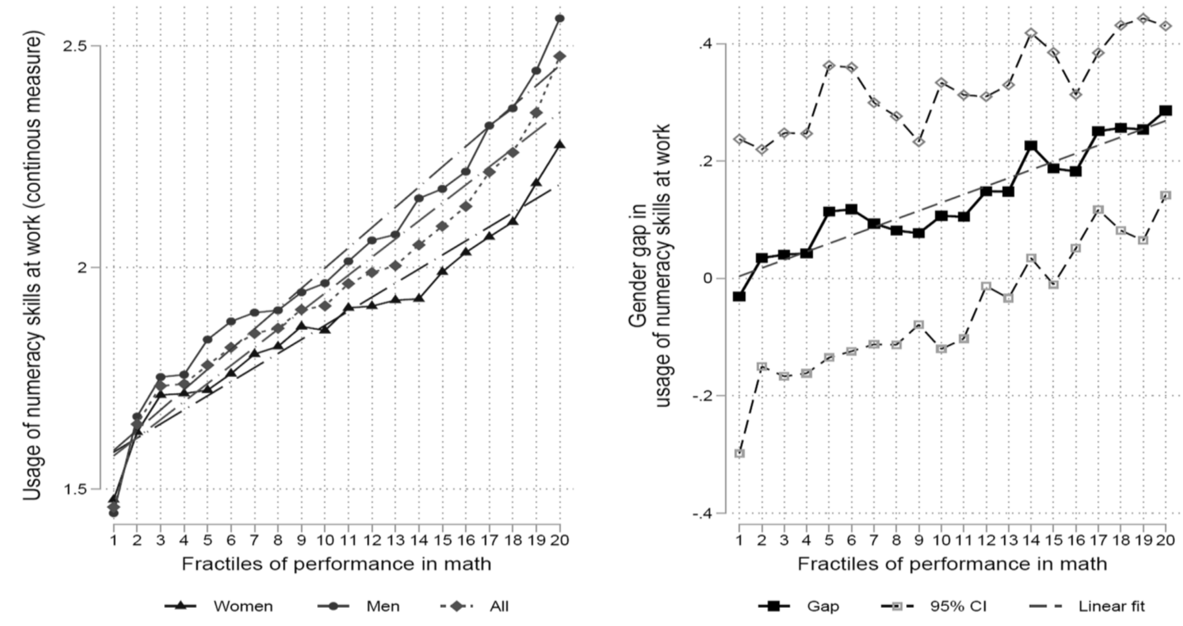

Supplementary Figures 9a to 9d are based on PIAAC data on about 150,000 adults aged 16-65 in 31 countries. Math performance is standardized to have a weighted mean equal to zero and a weighted standard deviation equal to one in each country in the sample. It is then split in 20 fractiles. Supplementary Figures 9a and 9b provides the proportion of females and males working in a math-related occupation (Supplementary Figure 9a) or in a STEM occupation (Supplementary Figure 9b) in each fractile. Supplementary Figure 9c provides for each fractile the proportion of females and males that use math intensively during their work. Supplementary Figure 9d provides for each fractile and for females and males the mean of an index capturing the extent to which working adults use math during their work. Confidence intervals are based on two-sided t-tests.

Supplementary Figure 10: Gender gap in comparative advantage for math and math self-concept, as a function of math performance

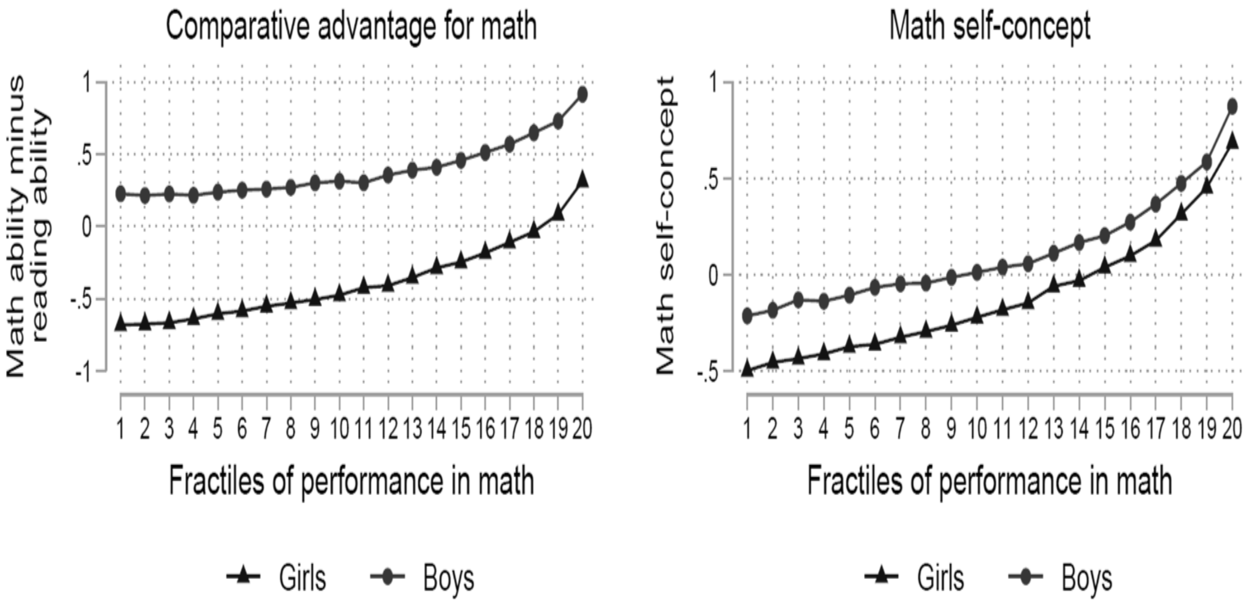

The Figure shows how girls' and boys' comparative advantage for math (math ability minus reading ability) and math self-concept vary with their math ability. See the legend of Figure 1 for details.

Supplementary Figure 11: Gender gap in students declared interest for math as a function of math performance

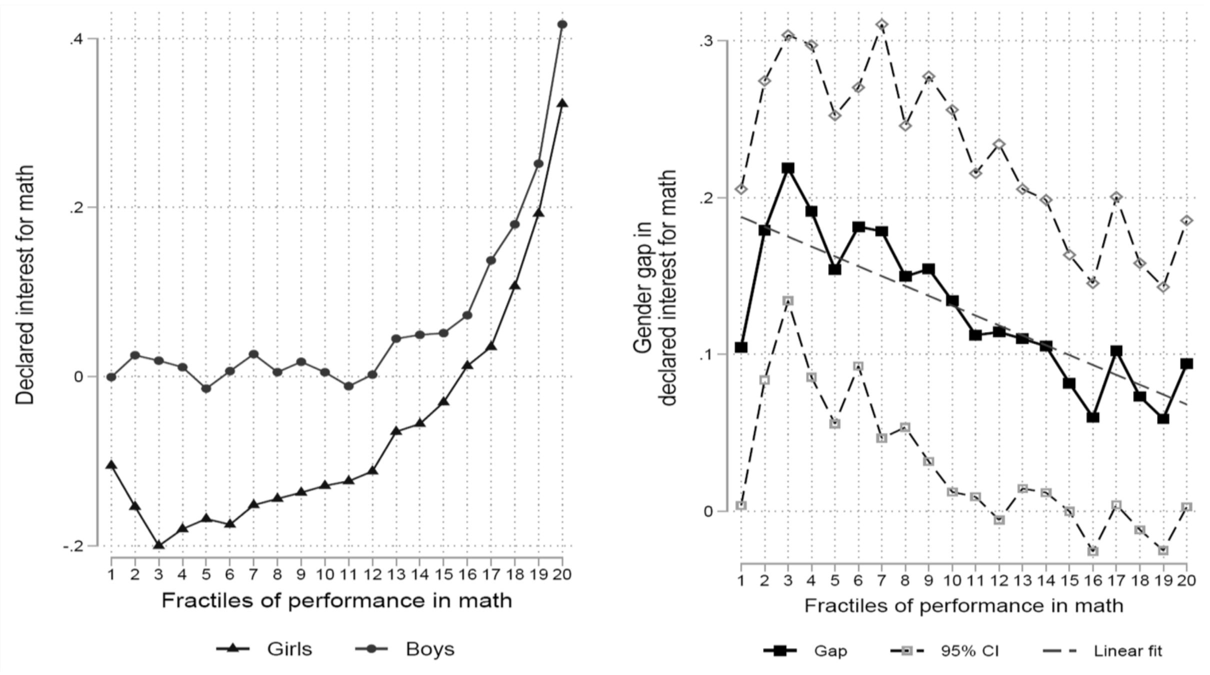

Declared interest for math is an index computed from several questions. It is standardized to have a mean equal to 0 and a standard deviation equal to 1 on the whole sample of students. Confidence intervals are based on two-sided t-tests.

Supplementary Figure 12: A non-parametric investigation of the relationship between math ability (raw measure rather than fractiles) and math intentions

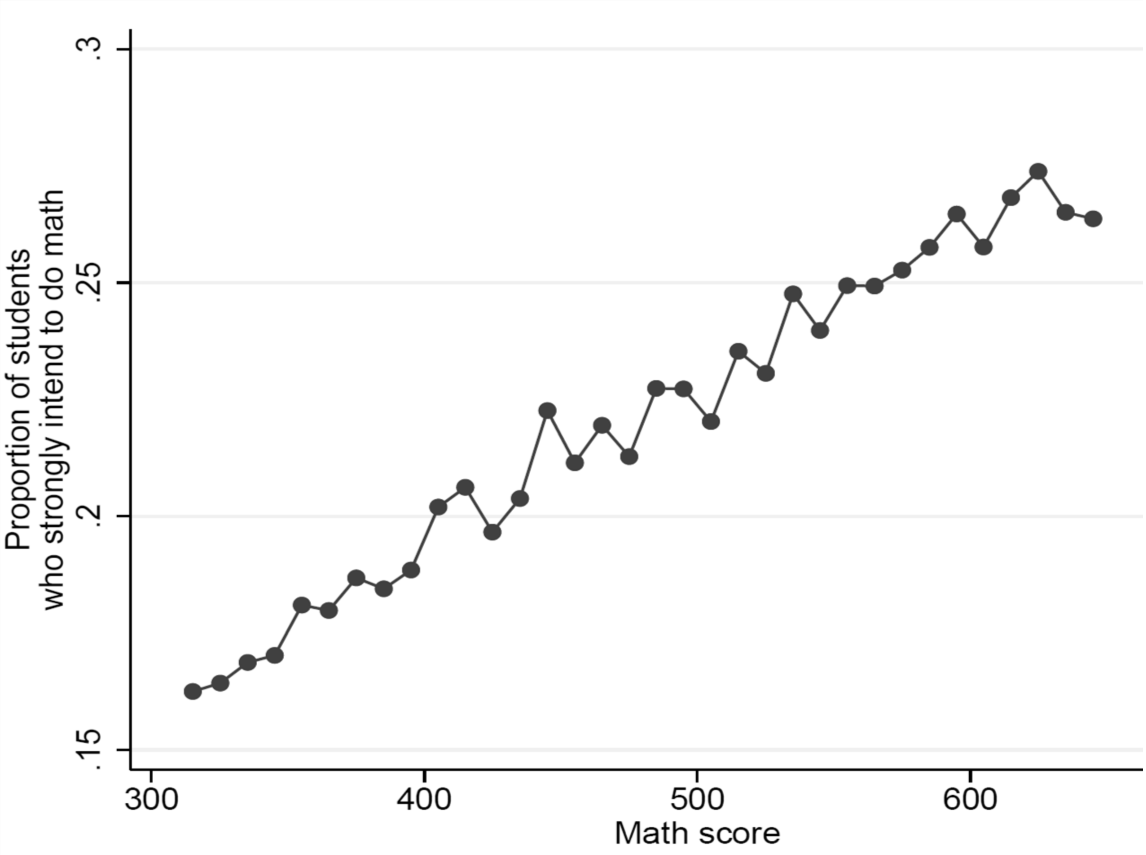

The Figure shows the proportion of students strongly intending to do math as a function of the math score (first plausible value only). Each dot shows the average score (x-axis) and the average proportion (y-axis) within a score bin of size 10 (e.g. all scores between 500 and 510). The bottom and top 5% of the score distribution have been excluded. The Figure shows that the relationship between the math score and intentions to do math is close to linear.

Supplementary Tables

Supplementary Table 1: Intentions to do math (PISA 2012) and graduates in STEM (UNESCO database), by country

	Percentage of students who strongly intend to do math				Percentage of graduates who graduate in STEM			
	All students	Girls	Boys	Boys minus Girls	All student s	Females	Males	Males minus Females
All PISA 2012 Countries	23,2	19,7	26,9	7,2				
OECD countries	22,0	17,5	26,5	9,0				
Non-OECD countries	24,5	22,0	27,3	5,2				
OECD countries:								
Australia	22,5	12,3	32,2	19,9	16,7	8,8	27,2	18,4
Austria	21,0	14,6	28,2	13,6	28,5	13,1	48,0	35,0
Belgium	21,1	15,1	27,7	12,6	16,7	6,8	30,9	24,1
Canada	18,0	10,4	25,8	15,4	20,7	11,6	33,6	22,0
Chile	25,4	18,3	33,2	15,0	19,5	7,0	35,5	28,5
Czech Republic	19,5	11,5	27,5	16,1	22,5	12,2	39,4	27,2
Denmark	30,4	24,1	36,9	12,9	21,3	13,8	31,5	17,7
Estonia	20,5	15,8	25,6	9,8	24,8	14,8	45,7	30,9
Finland	25,8	15,9	36,3	20,4	27,9	13,0	51,3	38,4
France	20,3	13,9	27,5	13,6	26,1	14,1	41,6	27,6
Germany	19,4	13,3	26,0	12,7	36,7	19,7	53,9	34,2
Greece	20,4	15,7	25,0	9,2	29,4	19,0	44,3	25,3
Hungary	19,6	14,1	25,6	11,5	18,6	8,8	36,1	27,2
Iceland	24,0	19,2	29,0	9,8	15,7	10,3	26,6	16,4
Ireland	16,9	12,3	21,4	9,1	25,1	13,5	39,0	25,5
Japan	19,0	15,8	22,0	6,2				
Korea	16,3	12,6	19,7	7,1	32,3	17,5	47,0	29,5
Luxembourg	20,6	14,3	27,1	12,7	14,7	10,4	20,7	10,2
Mexico	22,8	19,1	26,6	7,4	26,8	16,1	39,3	23,1
Netherlands	19,5	19,6	19,4	-0,2	13,8	6,0	24,2	18,2
New Zealand	18,2	12,0	24,4	12,4	19,2	11,0	31,2	20,2
Norway	32,8	25,7	40,2	14,4	16,3	8,3	29,1	20,8

Poland	25,3	18,5	33,2	14,7	18,1	11,2	31,5	20,4
Portugal	22,1	18,5	25,5	7,0	26,4	17,5	40,2	22,8
Slovak Republic	17,9	11,8	24,0	12,2	21,1	11,8	37,5	25,7
Slovenia	19,4	12,6	26,4	13,7	25,5	12,6	45,0	32,4
Spain	18,6	13,8	23,2	9,4	22,8	12,5	35,9	23,3
Sweden	31,8	25,2	38,9	13,7	27,3	14,7	47,0	32,3
Switzerland	24,0	13,5	35,7	22,2	21,8	9,5	33,1	23,6
Turkey	29,5	31,2	27,8	-3,4	21,2	15,6	26,3	10,7
United Kingdom	17,7	14,5	21,1	6,7	21,9	11,9	35,1	23,2
United States	23,4	18,3	28,5	10,1	16,6	8,8	27,5	18,7
Non -OECD countries:								
Albania	23,9	23,4	24,4	1,1	13,8	10,5	19,5	9,1
Argentina	21,6	17,1	26,7	9,6	13,6	9,1	21,9	12,7
Brazil	21,5	17,3	26,2	8,9	13,0	6,9	22,4	15,5
Bulgaria	17,2	13,6	20,6	7,0	22,7	15,0	34,0	19,0
Chinese Tapeï	17,1	17,2	17,0	-0,2				
Colombia	27,8	25,5	30,5	5,0	22,1	14,1	31,6	17,5
Costa Rica	27,2	22,3	32,6	10,3	12,4	6,4	23,1	16,7
Croatia	19,8	14,2	25,6	11,4	24,2	14,1	38,8	24,7
Hong Kong	13,9	10,8	16,6	5,8				
Indonesia	22,4	21,3	23,6	2,4	17,3	10,9	26,0	15,1
Jordan	20,9	19,1	23,0	3,9	26,4			
Kazakhstan	29,3	28,0	30,7	2,7	26,4	16,5	39,1	22,6
Latvia	20,6	15,7	25,5	9,8	20,0	9,5	41,9	32,3
Liechtenstein	28,0	21,8	34,5	12,6	24,9	33,8	21,1	-12,7
Macao	15,3	12,4	18,0	5,6	5,4	1,3	11,7	10,4
Malaysia	25,2	25,1	25,3	0,2	31,6	25,3	39,8	14,5
Montenegro	15,8	14,1	17,7	3,7				
Peru	29,6	25,3	34,0	8,6	23,5	14,0	35,6	21,6
Qatar	22,3	17,9	26,8	8,9	33,6	17,3	58,2	41,0
Romania	15,8	14,3	17,4	3,1	21,1	14,8	31,1	16,4
Russia	23,3	17,9	29,1	11,2	27,0			
Serbia	15,5	12,0	19,3	7,3	26,2	17,2	38,9	21,7
Shanghai-China	25,3	20,4	30,5	10,1				
Singapore	21,9	19,9	23,8	3,9	34,5	22,3	47,8	25,5
Thailand	25,0	25,7	24,1	-1,5	26,8	14,1	43,2	29,1

Tunisia	21,6	18,7	25,0	6,3	43,1	33,2	55,5	22,3
United Arab Emirates	18,3	16,3	20,4	4,1	26,2	20,7	33,9	13,3
Uruguay	26,9	25,3	29,0	3,8	15,6	10,8	24,2	13,4
Viet Nam	40,3	38,0	43,2	5,2	24,0	16,4	28,6	12,2

Notes: The proportion of students who graduate in STEM comes from the UNESCO database (http://data.uis.unesco.org/#, extracted on September 22nd, 2020) for years 2010-2016. When the information was missing for a country in 2012, the closest available year was considered instead. The proportion of students who strongly intend to do math corresponds to the proportion of students in PISA 2012 that answer yes to five questions measuring their intentions to pursue a career in a math-related field or to invest more in math than in other science subjects or reading/literature.

Supplementary Table 2 : Relationship between math ability and intentions to do math by gender and for various measures/standardizations of math ability and intentions to do math

Dependent variable:	*Dummy for strongly intending to do math*				*Index of intentions to do math*				*Index of intentions to do math standardized by country*	
	boys	girls	boys	girls	boys	girls	boys	girls	boys	girls
	(1)	(2)	(3)	(4)	(5)	(6)	(7)	(8)	(9)	(10)
Math performance (standardized by country)	.0541<br>[.0474, .0608]<br>p<.001	.0331<br>[.0276, .0386]<br>p<.001			.133<br>[.121, .145]<br>p<.001	.108<br>[.0959, .121]<br>p<.001			.132<br>[.119, .144]<br>p<.001	.106<br>[.0931, .118]<br>p<.001
Math performance (standardized worlwide)			.0446<br>[.0386, .0505]<br>p<.001	.0196<br>[.0145, .0247]<br>p<.001			.109<br>[.0978, .121]<br>p<.001	.0630<br>[.0501, .0759]<br>p<.001		
Constant	.262<br>[.255, .268]<br>p<.001	.197<br>[.192, .203]<br>p<.001	.263<br>[.257, .270]<br>p<.001	.197<br>[.192, .203]<br>p<.001	.102<br>[.0890, .116]<br>p<.001	-.0958<br>[-.109, -.0828]<br>p<.001	.105<br>[.0922, .119]<br>p<.001	-.0970<br>[-.110, -.0840]<br>p<.001	.0968<br>[.0836, .110]<br>p<.001	-.104<br>[-.116, -.0909]<br>p<.001
Observations	121,727	129,393	121,727	129,393	146,451	154,909	146,451	154,909	146,451	154,909
R-squared	.0153	.00635	.0105	.00227	.0177	.0107	.0120	.00366	.0177	.0106

Notes: The Table shows that boys rely more on their math performance to intend to study math (higher slopes and R-squared in all models) no matter how math performance and intentions to study math are standardized or measured. Analyses based on a sample of 61 countries. Math performance standardized by country is standardized to have a weighted mean equal to 0 and a weighted standard deviation equal to 1 in each country (and on the full sample of countries). Math performance standardized worldwide is an affine transformation of math ability to force the weighted mean to be equal to 0 and the weighted standard deviation to be equal to 1 on the full sample of countries. All estimates and standard errors are based on plausible values for math ability and account for measurement error in these abilities on top of standard sampling error. 95% Confidence intervals in brackets. P-values based on two-sided t-tests.

Supplementary Table 3: Differential intentions to do math of boys and girls (from Equation (1)). Controlling for country fixed effects and using alternative models

	Dependent variable is "strongly intending to do math"					
Model used:	**Linear probability**		**Logit**		**Probit**	
	(1)	(2)	(3)	(4)	(5)	(6)
Girl	-0.0645	-0.0665	-0.361	-0.378	-0.210	-0.221
	[-0.0718,-0.0571]	[-0.0738,-0.0592]	[-0.403,-0.318]	[-0.421,-0.335]	[-0.235,-0.185]	[-0.246,-0.196]
	p<0.001	p<0.001	p<0.001	p<0.001	p<0.001	p<0.001
Math performance	0.0541	0.0548	0.277	0.286	0.166	0.170
	[0.0474,0.0608]	[0.0481,0.0616]	[0.243,0.312]	[0.250,0.321]	[0.145,0.187]	[0.149,0.191]
	p<0.001	p<0.001	p<0.001	p<0.001	p<0.001	p<0.001
Girl*(math performance)	-0.0210	-0.0218	-0.0690	-0.0752	-0.0470	-0.0501
	[-0.0295,-0.0125]	[-0.0305,-0.0131]	[-0.117,-0.0209]	[-0.125,-0.0256]	[-0.0752,-0.0188]	[-0.0791,-0.0212]
	p<0.001	p<0.001	p=0.005	p=0.003	p=0.001	p=0.001
Country fixed effects	No	Yes	No	Yes	No	Yes
Observations	251,120	251,120	251,120	251,120	251,120	251,120

Notes: Analyses based on a sample of 61 countries. Math performance is standardized to have a weighted mean equal to 0 and a weighted standard deviation equal to 1 in each country of the sample (and on the full sample of countries). "Strongly intending to do math" corresponds to answering yes to five questions measuring PISA 2012 students' intentions to pursue a career in a math-related field or to invest more in math than in other science subjects or reading/literature. All estimates and standard errors are based on plausible values for math ability and account for measurement error in these abilities on top of standard sampling error. 95% confidence intervals in brackets. P-values based on two-sided t-tests.

Supplementary Table 4: The role of math ability to explain math intentions: boys versus girls

	Estimates from the regression $Math\_intention_i = \alpha Girl_i + \beta Math\_ability_i + \gamma(Math\_ability_i * Girl_i) + \varepsilon_i$				Coefficient β in the regression $Math\_intention_i = \beta Math\_ability_i + +\varepsilon_i$ applied to both girls and boys
	**Gender gap in math intentions for average ability students' (α)**	**Math ability and math intentions for boys (β)**	**Gender gap (girls minus boys) in the regression of math intentions on math ability (γ)**	**Ratio of "slopes" $(\beta + \gamma) / \beta$**	
All PISA 2012 Countries	-0.064	0.054	-0.021	0,61	0.046
	[-0.072,-0.057]	[0.047,0.061]	[-0.030,-0.013]		[0.042,0.051]
	p<.001	p<.001	p<.001		p<.001
All OECD countries	-0.083	0.044	-0.019	0,57	0.038
	[-0.094,-0.073]	[0.036,0.052]	[-0.031,-0.006]		[0.032,0.044]
	p<.001	p<.001	p=0.004		p<.001
All Non-OECD countries	-0.045	0.066	-0.025	0,62	0.055
	[-0.055,-0.034]	[0.055,0.078]	[-0.036,-0.014]		[0.047,0.063]
	p<.001	p<.001	p<.001		p<.001
OECD countries (ordered by decreasing order of the slope of the relationship between math ability and math intention for boys)					
Poland	-0.129	0.171	-0.057	0,67	0.146
	[-0.162,-0.095]	[0.146,0.195]	[-0.092,-0.022]		[0.129,0.164]
	p<.001	p<.001	p=0.002		p<.001
Hungary	-0.097	0.133	-0.064	0,52	0.106
	[-0.128,-0.067]	[0.105,0.160]	[-0.096,-0.033]		[0.087,0.125]
	p<.001	p<.001	p<.001		p<.001
Finland	-0.188	0.129	-0.066	0,49	0.101
	[-0.214,-0.161]	[0.107,0.151]	[-0.096,-0.036]		[0.085,0.117]
	p<.001	p<.001	p<.001		p<.001
Slovak Republic	-0.107	0.119	-0.057	0,52	0.098
	[-0.135,-0.079]	[0.091,0.147]	[-0.092,-0.021]		[0.079,0.116]
	p<.001	p<.001	p=0.002		p<.001
Norway	-0.135	0.085	-0.021	0,75	0.078
	[-0.177,-0.094]	[0.058,0.113]	[-0.063,0.020]		[0.060,0.097]
	p<.001	p<.001	p=0.313		p<.001
Luxembourg	-0.107	0.084	-0.059	0,30	0.064
	[-0.137,-0.076]	[0.061,0.108]	[-0.090,-0.028]		[0.050,0.078]
	p<.001	p<.001	p<.001		p<.001
Czech Republic	-0.142	0.082	-0.047	0,43	0.070

	[-0.181,-0.104]	[0.055,0.110]	[-0.086,-0.008]		[0.052,0.088]
	p<.001	p<.001	p=0.019		p<.001
Estonia	-0.088	0.081	-0.036	0,55	0.066
	[-0.118,-0.058]	[0.055,0.106]	[-0.068,-0.004]		[0.050,0.083]
	p<.001	p<.001	p=0.027		p<.001
Greece	-0.082	0.079	-0.053	0,33	0.059
	[-0.115,-0.049]	[0.057,0.102]	[-0.085,-0.021]		[0.043,0.074]
	p<.001	p<.001	p=0.001		p<.001
Chile	-0.127	0.076	-0.02	0,73	0.076
	[-0.159,-0.095]	[0.053,0.100]	[-0.051,0.010]		[0.061,0.092]
	p<.001	p<.001	p=0.194		p<.001
Sweden	-0.131	0.068	-0.024	0,64	0.058
	[-0.170,-0.092]	[0.038,0.098]	[-0.067,0.018]		[0.038,0.078]
	p<.001	p<.001	p=0.255		p<.001
Belgium	-0.116	0.065	-0.03	0,54	0.054
	[-0.144,-0.087]	[0.044,0.086]	[-0.058,-0.002]		[0.040,0.068]
	p<.001	p<.001	p=0.038		p<.001
Spain	-0.08	0.061	-0.045	0,27	0.046
	[-0.101,-0.059]	[0.044,0.078]	[-0.067,-0.022]		[0.036,0.057]
	p<.001	p<.001	p<.001		p<.001
Mexico	-0.065	0.06	-0.025	0,59	0.051
	[-0.080,-0.051]	[0.048,0.073]	[-0.042,-0.008]		[0.042,0.061]
	p<.001	p<.001	p=0.005		p<.001
Slovenia	-0.129	0.059	-0.042	0,30	0.043
	[-0.165,-0.092]	[0.032,0.087]	[-0.077,-0.006]		[0.024,0.062]
	p<.001	p<.001	p=0.021		p<.001
Korea	-0.062	0.057	-0.025	0,56	0.050
	[-0.087,-0.036]	[0.040,0.075]	[-0.048,-0.003]		[0.037,0.063]
	p<.001	p<.001	p=0.028		p<.001
Turkey	0.04	0.054	-0.012	0,77	0.046
	[0.001,0.079]	[0.029,0.079]	[-0.050,0.025]		[0.026,0.067]
	p=0.043	p<.001	p=0.510		p<.001
Austria	-0.12	0.043	-0.014	0,67	0.048
	[-0.156,-0.085]	[0.012,0.074]	[-0.058,0.030]		[0.030,0.067]
	p<.001	p=0.006	p=0.528		p<.001
United States	-0.097	0.04	-0.027	0,31	0.029
	[-0.129,-0.066]	[0.014,0.065]	[-0.065,0.010]		[0.012,0.046]
	p<.001	p=0.002	p=0.156		p<.001
Switzerland	-0.213	0.04	-0.037	0,06	0.031
	[-0.253,-0.174]	[0.015,0.064]	[-0.069,-0.005]		[0.017,0.045]
	p<.001	p=0.002	p=0.024		p<.001
New Zealand	-0.118	0.035	-0.054	-0,55	0.016
	[-0.149,-0.086]	[0.013,0.056]	[-0.087,-0.020]		[-0.004,0.035]
	p<.001	p=0.002	p=0.002		p=0.122
France	-0.129	0.03	-0.024	0,19	0.025
	[-0.172,-0.085]	[-0.004,0.063]	[-0.066,0.018]		[0.004,0.046]
	p<.001	p=0.084	p=0.263		p=0.018

Portugal	-0.066	0.026	-0.018	0,30	0.020
	[-0.094,-0.038]	[0.004,0.048]	[-0.048,0.011]		[0.004,0.037]
	p<.001	p=0.019	p=0.221		p=0.017
Iceland	-0.101	0.024	0.033	2,40	0.039
	[-0.143,-0.058]	[-0.007,0.054]	[-0.004,0.071]		[0.017,0.062]
	p<.001	p=0.126	p=0.082		p<.001
Ireland	-0.087	0.023	-0.018	0,23	0.019
	[-0.115,-0.060]	[-0.004,0.050]	[-0.054,0.019]		[0.002,0.037]
	p<.001	p=0.097	p=0.343		p=0.030
Japan	-0.058	0.023	-0.008	0,63	0.023
	[-0.081,-0.034]	[0.002,0.043]	[-0.038,0.022]		[0.009,0.036]
	p<.001	p=0.030	p=0.586		p<.001
Denmark	-0.125	0.017	0.019	2,12	0.033
	[-0.161,-0.089]	[-0.015,0.048]	[-0.023,0.060]		[0.013,0.052]
	p<.001	p=0.293	p=0.372		p=0.001
Canada	-0.151	0.014	0.005	1,35	0.023
	[-0.171,-0.131]	[-0.005,0.033]	[-0.019,0.028]		[0.012,0.034]
	p<.001	p=0.150	p=0.686		p<.001
United Kingdom	-0.065	0.01	0.009	1,83	0.017
	[-0.093,-0.038]	[-0.014,0.035]	[-0.030,0.047]		[0.004,0.031]
	p<.001	p=0.409	p=0.663		p=0.013
Germany	-0.128	0.008	0.031	4,83	0.030
	[-0.163,-0.094]	[-0.021,0.037]	[-0.007,0.069]		[0.010,0.050]
	p<.001	p=0.581	p=0.106		p=0.003
Netherlands	0.001	-0.007	0.005		-0.004
	[-0.035,0.037]	[-0.031,0.018]	[-0.027,0.036]		[-0.023,0.014]
	p=0.947	p=0.591	p=0.762		p=0.636
Australia	-0.203	-0.017	0.025		0.002
	[-0.221,-0.184]	[-0.034,-0.000]	[0.003,0.047]		[-0.009,0.013]
	p<.001	p=0.044	p=0.025		p=0.710
Non-OECD countries (ordered by decreasing order of the slope of the relationship between math ability and math intention for boys)					
Russia	-0.107	0.136	-0.064	0,53	0.104
	[-0.130,-0.084]	[0.110,0.161]	[-0.092,-0.036]		[0.083,0.125]
	p<.001	p<.001	p<.001		p<.001
Tunisia	-0.037	0.102	-0.008	0,92	0.100
	[-0.071,-0.003]	[0.072,0.132]	[-0.045,0.029]		[0.080,0.120]
	p=0.035	p<.001	p=0.682		p<.001
Romania	-0.025	0.096	-0.036	0,62	0.078
	[-0.053,0.002]	[0.073,0.119]	[-0.066,-0.006]		[0.063,0.094]
	p=0.071	p<.001	p=0.017		p<.001
Bulgaria	-0.066	0.093	-0.023	0,75	0.083
	[-0.094,-0.039]	[0.069,0.117]	[-0.055,0.010]		[0.065,0.101]
	p<.001	p<.001	p=0.167		p<.001
Latvia	-0.102	0.089	-0.034	0,61	0.072
	[-0.138,-0.065]	[0.062,0.116]	[-0.074,0.005]		[0.055,0.089]
	p<.001	p<.001	p=0.090		p<.001
Thailand	0.011	0.088	-0.055	0,37	0.058

	[-0.018,0.039]	[0.064,0.111]	[-0.086,-0.024]		[0.041,0.074]
	p=0.462	p<.001	p<.001		p<.001
Peru	-0.07	0.077	-0.036	0,53	0.062
	[-0.102,-0.038]	[0.051,0.102]	[-0.071,-0.001]		[0.043,0.082]
	p<.001	p<.001	p=0.041		p<.001
Viet Nam	-0.044	0.065	0.013	1,20	0.073
	[-0.082,-0.006]	[0.035,0.095]	[-0.022,0.048]		[0.045,0.101]
	p=0.022	p<.001	p=0.473		p<.001
Kazakhstan	-0.026	0.064	-0.047	0,26	0.042
	[-0.059,0.007]	[0.037,0.091]	[-0.086,-0.009]		[0.020,0.063]
	p=0.124	p<.001	p=0.017		p<.001
Colombia	-0.031	0.062	-0.038	0,39	0.046
	[-0.068,0.005]	[0.036,0.087]	[-0.080,0.005]		[0.029,0.063]
	p=0.092	p<.001	p=0.080		p<.001
Uruguay	-0.026	0.06	0	1,00	0.061
	[-0.062,0.009]	[0.033,0.088]	[-0.044,0.043]		[0.041,0.082]
	p=0.148	p<.001	p=0.998		p<.001
Brazil	-0.078	0.06	-0.029	0,51	0.049
	[-0.099,-0.057]	[0.041,0.079]	[-0.052,-0.007]		[0.037,0.061]
	p<.001	p<.001	p=0.011		p<.001
Argentina	-0.083	0.058	-0.029	0,51	0.049
	[-0.115,-0.051]	[0.027,0.089]	[-0.065,0.008]		[0.028,0.070]
	p<.001	p<.001	p=0.122		p<.001
Serbia	-0.063	0.056	-0.004	0,94	0.057
	[-0.094,-0.032]	[0.028,0.084]	[-0.039,0.032]		[0.041,0.074]
	p<.001	p<.001	p=0.842		p<.001
Malaysia	-0.003	0.054	-0.05	0,08	0.028
	[-0.033,0.027]	[0.028,0.080]	[-0.084,-0.016]		[0.009,0.048]
	p=0.852	p<.001	p=0.004		p=0.005
Montenegro	-0.035	0.053	-0.003	0,94	0.052
	[-0.066,-0.003]	[0.023,0.084]	[-0.043,0.037]		[0.034,0.070]
	p=0.030	p<.001	p=0.867		p<.001
Chinese Tapeï	0.006	0.047	0.003	1,06	0.049
	[-0.021,0.033]	[0.029,0.065]	[-0.028,0.034]		[0.035,0.062]
	p=0.660	p<.001	p=0.851		p<.001
Indonesia	-0.018	0.047	-0.007	0,85	0.044
	[-0.050,0.014]	[0.009,0.084]	[-0.048,0.034]		[0.018,0.070]
	p=0.261	p=0.015	p=0.743		p<.001
Croatia	-0.104	0.046	-0.028	0,40	0.039
	[-0.138,-0.070]	[0.024,0.069]	[-0.062,0.006]		[0.022,0.057]
	p<.001	p<.001	p=0.110		p<.001
Shanghai-China	-0.098	0.032	0.005	1,16	0.036
	[-0.132,-0.065]	[0.012,0.052]	[-0.024,0.034]		[0.025,0.048]
	p<.001	p=0.002	p=0.733		p<.001
United Arab Emirates	-0.04	0.027	-0.019	0,32	0.019
	[-0.062,-0.019]	[0.012,0.043]	[-0.041,0.003]		[0.009,0.029]

	p<.001	p<.001	p=0.099		p<.001
Hong Kong	-0.054	0.023	-0.007	0,71	0.023
	[-0.074,-0.033]	[0.004,0.042]	[-0.031,0.018]		[0.010,0.036]
	p<.001	p=0.016	p=0.582		p<.001
Liechtenstein	-0.119	0.016	0.002	1,14	0.031
	[-0.262,0.024]	[-0.111,0.144]	[-0.150,0.155]		[-0.039,0.101]
	p=0.105	p=0.801	p=0.976		p=0.382
Qatar	-0.088	0.012	-0.008	0,31	0.006
	[-0.113,-0.063]	[-0.006,0.029]	[-0.032,0.016]		[-0.004,0.017]
	p<.001	p=0.193	p=0.521		p=0.227
Costa Rica	-0.107	-0.002	-0.021	NA	-0.002
	[-0.143,-0.070]	[-0.030,0.026]	[-0.069,0.028]		[-0.025,0.021]
	p<.001	p=0.881	p=0.401		p=0.849
Albania	-0.011	-0.003	-0.012	NA	-0.009
	[-0.060,0.038]	[-0.033,0.027]	[-0.056,0.031]		[-0.029,0.010]
	p=0.670	p=0.846	p=0.577		p=0.342
Macao	-0.056	-0.012	0.014	NA	-0.005
	[-0.082,-0.031]	[-0.030,0.007]	[-0.010,0.038]		[-0.017,0.007]
	p<.001	p=0.219	p=0.239		p=0.449
Singapore	-0.039	-0.012	-0.005	NA	-0.014
	[-0.062,-0.016]	[-0.033,0.009]	[-0.035,0.026]		[-0.028,0.000]
	p<.001	p=0.267	p=0.764		p=0.056
Jordan	-0.035	-0.019	-0.007	NA	-0.024
	[-0.069,-0.001]	[-0.044,0.006]	[-0.041,0.027]		[-0.042,-0.005]
	p=0.043	p=0.143	p=0.683		p=0.012

Notes: The Table provides estimates from Equation (1) without control variables for all countries in the PISA 2012 sample. a: In these countries, intentions to pursue math-related studies or careers do not increase significantly (at the 5% level) with math performance (in a regression that does not distinguish between girls and boys). 95% Confidence intervals in brackets. P-values based on two-sided t-tests. Math ability is standardized to have a weighted mean equal to 0 and a weighted standard deviation equal to 1 in each country. Math intention is a dummy equal to one for students answering yes to a series of 5 questions capturing intentions to pursue studies or careers related to math. Estimates and standard errors involving are based on plausible values for math ability and account for measurement error in these abilities on top of standard sampling error.

Supplementary Table 5: Proportion of students and ratio of girls and boys intending to do math by quartile of math performance

quartile:	First	Second	Third	Fourth
ALL	17.3%	21.0%	24.0%	29.4%
Boys	18.8%	24.3%	27.9%	33.8%
Girls	15.9%	18.3%	20.5%	24.2%
Gender Gap	2.9%	6.0%	7.4%	9.6%
Boys-to-girls ratio	1.18	1.33	1.37	1.41

Notes: Based on 61 countries in PISA 2012. See details in other Tables.

Supplementary Table 6: Comparing gender gaps in math performance among those strongly intending to pursue math studies and in the whole population (all countries)

	a) Gender gap (girls minus boys) in the whole population	b) Gender gap among those strongly intending to do math	c) Ratio of Gender gaps (a/b)	d) Girls-to-boys ratio in top decile of whole population	e) Girls-to-boys ratio in top decile of those strongly intending to do math
OECD countries					
Australia	-0.157<br>[-0.229,-0.084]<br>p<.001	-0.051<br>[-0.174,0.073]<br>p=0.421	0.323	0.591	0.336
Austria	-0.370<br>[-0.477,-0.263]<br>p<.001	-0.345<br>[-0.556,-0.134]<br>p=0.001	0.932	0.441	0.156
Belgium	-0.131<br>[-0.210,-0.052]<br>p=0.001	-0.149<br>[-0.311,0.013]<br>p=0.072	1.139	0.789	0.456
Canada	-0.159<br>[-0.216,-0.102]<br>p<.001	-0.055<br>[-0.192,0.082]<br>p=0.430	0.347	0.646	0.424
Chile	-0.311<br>[-0.406,-0.216]<br>p<.001	-0.261<br>[-0.404,-0.118]<br>p<.001	0.840	0.606	0.416
Czech Republic	-0.240<br>[-0.343,-0.138]<br>p<.001	-0.246<br>[-0.476,-0.017]<br>p=0.036	1.026	0.650	0.332
Denmark	-0.189<br>[-0.267,-0.111]<br>p<.001	-0.097<br>[-0.250,0.056]<br>p=0.214	0.516	0.682	0.490
Estonia	-0.131<br>[-0.204,-0.059]<br>p<.001	-0.198<br>[-0.369,-0.026]<br>p=0.024	1.503	0.715	0.364
Finland	-0.083<br>[-0.161,-0.004]<br>p=0.039	-0.087<br>[-0.231,0.057]<br>p=0.237	1.051	0.799	0.366
France	-0.206<br>[-0.296,-0.116]<br>p<.001	-0.281<br>[-0.504,-0.057]<br>p=0.014	1.361	0.608	0.321
Germany	-0.211<br>[-0.310,-0.112]<br>p<.001	0.011<br>[-0.209,0.232]<br>p=0.920	-0.054	0.622	0.624
Greece	-0.140<br>[-0.234,-0.046]<br>p=0.004	-0.333<br>[-0.518,-0.148]<br>p<.001	2.381	0.635	0.315
Hungary	-0.137<br>[-0.230,-0.043]<br>p=0.004	-0.256<br>[-0.441,-0.072]<br>p=0.007	1.875	0.713	0.391
Iceland	-0.002<br>[-0.096,0.092]<br>p=0.968	0.164<br>[0.007,0.321]<br>p=0.041	-84.762	0.842	0.746
Ireland	-0.218	-0.282	1.294	0.588	0.300

	[-0.316,-0.119] p<.001	[-0.531,-0.033] p=0.027			
Japan	-0.230 [-0.321,-0.139] p<.001	-0.260 [-0.427,-0.094] p=0.002	1.133	0.470	0.311
Korea	-0.212 [-0.346,-0.079] p=0.002	-0.317 [-0.516,-0.118] p=0.002	1.494	0.384	0.170
Luxembourg	-0.301 [-0.367,-0.236] p<.001	-0.451 [-0.616,-0.286] p<.001	1.497	0.534	0.153
Mexico	-0.168 [-0.206,-0.130] p<.001	-0.233 [-0.318,-0.149] p<.001	1.388	0.694	0.457
Netherlands	-0.176 [-0.238,-0.113] p<.001	-0.153 [-0.308,0.001] p=0.053	0.871	0.620	0.489
New Zealand	-0.177 [-0.274,-0.081] p<.001	-0.465 [-0.710,-0.219] p<.001	2.619	0.605	0.219
Norway	-0.083 [-0.154,-0.013] p=0.021	-0.077 [-0.224,0.069] p=0.301	0.928	0.812	0.624
Poland	-0.115 [-0.215,-0.015] p=0.024	-0.108 [-0.275,0.059] p=0.205	0.944	0.736	0.400
Portugal	-0.155 [-0.223,-0.088] p<.001	-0.222 [-0.377,-0.067] p=0.005	1.431	0.621	0.393
Slovak Republic	-0.126 [-0.235,-0.018] p=0.022	-0.214 [-0.419,-0.010] p=0.040	1.697	0.483	0.204
Slovenia	-0.126 [-0.212,-0.040] p=0.004	-0.210 [-0.432,0.012] p=0.064	1.669	0.812	0.447
Spain	-0.275 [-0.331,-0.219] p<.001	-0.438 [-0.569,-0.306] p<.001	1.593	0.457	0.214
Sweden	-0.053 [-0.144,0.037] p=0.248	-0.079 [-0.213,0.055] p=0.247	1.480	0.780	0.469
Switzerland	-0.176 [-0.248,-0.103] p<.001	-0.267 [-0.424,-0.110] p<.001	1.519	0.758	0.273
Turkey	-0.122 [-0.237,-0.007] p=0.038	-0.190 [-0.333,-0.047] p=0.009	1.561	0.752	0.731
United Kingdom	-0.146 [-0.252,-0.040] p=0.007	-0.073 [-0.296,0.149] p=0.518	0.504	0.795	0.845
United States	-0.109 [-0.182,-0.035] p=0.004	-0.191 [-0.359,-0.022] p=0.027	1.756	0.787	0.582
Non-OECD countries					

Albania	0.011 [-0.086,0.109] p=0.820	-0.045 [-0.268,0.179] p=0.695	-3.937	0.994	0.795
Argentina	-0.240 [-0.346,-0.134] p<.001	-0.303 [-0.501,-0.105] p=0.003	1.262	0.725	0.391
Brazil	-0.194 [-0.253,-0.136] p<.001	-0.272 [-0.383,-0.162] p<.001	1.399	0.732	0.402
Bulgaria	-0.026 [-0.120,0.067] p=0.583	-0.034 [-0.232,0.164] p=0.736	1.301	0.691	0.493
Chinese Tapeï	-0.090 [-0.246,0.067] p=0.261	-0.116 [-0.253,0.021] p=0.097	1.293	0.743	0.515
Colombia	-0.344 [-0.453,-0.235] p<.001	-0.467 [-0.655,-0.280] p<.001	1.358	0.517	0.324
Costa Rica	-0.367 [-0.464,-0.269] p<.001	-0.450 [-0.644,-0.256] p<.001	1.227	0.509	0.274
Croatia	-0.215 [-0.323,-0.107] p<.001	-0.294 [-0.510,-0.077] p=0.008	1.367	0.579	0.279
Hong Kong	-0.184 [-0.313,-0.056] p=0.005	-0.197 [-0.395,0.002] p=0.053	1.068	0.469	0.205
Indonesia	-0.114 [-0.227,-0.002] p=0.047	-0.136 [-0.338,0.066] p=0.189	1.185	0.801	0.948
Jordan	0.147 [-0.012,0.306] p=0.070	0.131 [-0.078,0.341] p=0.219	0.892	1.211	0.877
Kazakhstan	-0.011 [-0.111,0.088] p=0.823	-0.176 [-0.343,-0.009] p=0.039	15.485	0.882	0.627
Latvia	0.053 [-0.050,0.156] p=0.311	0.001 [-0.219,0.221] p=0.991	0.025	0.864	0.512
Liechtenstein	-0.427 [-0.702,-0.153] p=0.003	-0.376 [-0.901,0.150] p=0.169	0.879	0.350	.
Macao	-0.024 [-0.081,0.034] p=0.418	0.061 [-0.089,0.212] p=0.422	-2.585	0.836	0.630
Malaysia	0.070 [-0.030,0.171] p=0.171	-0.135 [-0.261,-0.009] p=0.036	-1.913	1.178	0.748
Montenegro	-0.031 [-0.114,0.053] p=0.474	-0.006 [-0.244,0.232] p=0.960	0.198	1.081	0.678
Peru	-0.204 [-0.305,-0.103] p<.001	-0.272 [-0.403,-0.142] p<.001	1.336	0.758	0.484

Qatar	0.068 [0.021,0.114] p=0.005	0.038 [-0.078,0.155] p=0.519	0.566	0.870	0.593
Romania	-0.048 [-0.149,0.052] p=0.346	-0.221 [-0.420,-0.022] p=0.030	4.576	0.916	0.594
Russia	-0.017 [-0.085,0.052] p=0.638	-0.097 [-0.254,0.059] p=0.223	5.896	1.040	0.625
Serbia	-0.170 [-0.265,-0.074] p<.001	-0.037 [-0.268,0.193] p=0.751	0.220	0.804	0.620
Shanghai-China	-0.075 [-0.148,-0.002] p=0.045	-0.014 [-0.144,0.116] p=0.832	0.187	0.835	0.628
Singapore	0.013 [-0.048,0.074] p=0.679	-0.009 [-0.151,0.134] p=0.905	-0.668	0.805	0.674
Thailand	0.104 [0.011,0.197] p=0.029	-0.149 [-0.295,-0.003] p=0.046	-1.431	1.364	0.866
Tunisia	-0.262 [-0.343,-0.181] p<.001	-0.196 [-0.357,-0.035] p=0.017	0.750	0.750	0.715
United Arab Emirates	0.012 [-0.087,0.112] p=0.810	-0.093 [-0.239,0.052] p=0.210	-7.644	0.750	0.452
Uruguay	-0.191 [-0.270,-0.111] p<.001	-0.203 [-0.374,-0.031] p=0.021	1.063	0.705	0.604
Viet Nam	-0.124 [-0.205,-0.042] p=0.003	-0.101 [-0.203,0.001] p=0.052	0.819	0.805	0.644

Notes: 95% Confidence intervals in brackets. P-values based on two-sided t-tests. Math ability is standardized to have a weighted mean equal to 0 and a weighted standard deviation equal to 1 in each country. Gender gaps are therefore expressed as a fraction of the standard deviation of math performance among the whole population. "Strongly intending to do math" corresponds to answering yes to five questions measuring PISA 2012 students' intentions to pursue a career in a math-related field or to invest more in math than in other science subjects or reading/literature. The last two columns provide the ratio between the number of girls and boys in the top decile of the ability distribution both in the whole population and among those strongly intending to pursue math studies. All estimates and standard errors are based on plausible values for math ability and account for measurement error in these abilities on top of standard sampling error.

Supplementary Table 7: Comparing gender gaps in math performance among those strongly intending to do math and in the whole population

	a) Gender gap (girls minus boys) in the whole population	b) Gender gap among those strongly intending to do math	c) Increase in gender gap due to intentions (a-b)/b	d) Girls-to-boys ratio in whole population	e) Girls-to-boys ratio among those strongly intending to do math	f) Girls-to-boys ratio within top decile of math performance	g) Girls-to-boys ratio among those strongly intending to do math within top decile of math performance
Panel A: Distribution of math performance of girls and boys taken as given (not reweighted)							
All PISA 2012 Countries	-0.137 [-0.157,-0.116] p<.001	-0.190 [-0.230,-0.151] p<.001	38,90%	1.060	0.777	0.743	0.536
OECD countries	-0.158 [-0.186,-0.131] p<.001	-0.199 [-0.260,-0.138] p<.001	25,70%	1.060	0.777	0.671	0.482
Non-OECD countries	-0.113 [-0.146,-0.081] p<.001	-0.191 [-0.240,-0.141] p<.001	68,50%	1.020	0.673	0.832	0.596
Panel B: Distribution of math performance of girls and boys in whole population reweighted to make them equal							
All PISA 2012 Countries	-0.000 [-0.008,0.008] p=0.940	-0.037 [-0.054,-0.020] p<.001		1.060	0.796	1.060	0.790
OECD countries	0.000 [-0.010,0.010] p=0.957	-0.017 [-0.040,0.005] p=0.326		1.020	0.690	1.019	0.684
Non-OECD countries	-0.001 [-0.013,0.012] p=0.913	-0.069 [-0.095,-0.044] p<.001		1.107	0.912	1.107	0.904

Notes: 95% Confidence intervals in brackets. P-values based on two-sided t-tests. Math ability is standardized to have a weighted mean equal to 0 and a weighted standard deviation equal to 1 in each country. Gender gaps are therefore expressed as a fraction of the standard deviation of math performance among the whole population. "Strongly intending to do math" corresponds to answering yes to five questions measuring PISA 2012 students' intentions to pursue a career in a math-related field or to invest more in math than in other science subjects or reading/literature. The last two columns provide the ratio between the number of girls and boys in the top decile of the ability distribution both in the whole population and among those strongly intending to pursue math studies. All estimates and standard errors are based on plausible values for math ability and account for measurement error in these abilities on top of standard sampling error.

Supplementary Table 8: Differential intentions to do math (from Equation (1)) for various measures of intentions

	(1)	(2)	(3)	(4)	(5)	(6)	(7)	(8)
Dependent variable:	Dummy for strongly intending to do math (main measure)	Dummy for strongly intending to do math (main measure)	Continuous index measuring intentions to do math	Continuous index measuring intentions to do math standardized by country	Dummy for being in top 25% of intentions to do math	Dummy for being in top 10% of intentions to do math	Favoring math over science	Favoring math over reading
Girl	-.0645<br>[-.0718, -.0571]<br>p<.001	-.0662<br>[-.0736, -.0588]<br>p<.001	-.198<br>[-.216, -.181]<br>p<.001	-.200<br>[-.218, -.183]<br>p<.001	-.0825<br>[-.0900, -.0749]<br>p<.001	-.0569<br>[-.0635, -.0502]<br>p<.001	-.0569<br>[-.0662, -.0477]<br>p<.001	-.0599<br>[-.0679, -.0519]<br>p<.001
Math performance	.0541<br>[.0474, .0608]<br>p<.001	.0446<br>[.0386, .0505]<br>p<.001	.133<br>[.121, .145]<br>p<.001	.132<br>[.119, .144]<br>p<.001	.0469<br>[.0405, .0533]<br>p<.001	.0531<br>[.0471, .0591]<br>p<.001	.0467<br>[.0395, .0538]<br>p<.001	.110<br>[.105, .116]<br>p<.001
**Girl*(Math performance)**	-.0210<br>[-.0295, -.0125]<br>p<.001	-.0250<br>[-.0324, -.0175]<br>p<.001	-.0246<br>[-.0424, -.00672]<br>p=.007	-.0261<br>[-.0441, -.00802]<br>p=.005	-.0205<br>[-.0295, -.0115]<br>p<.001	-.0197<br>[-.0275, -.0119]<br>p<.001	-.0178<br>[-.0276, -.00807]<br>p<.001	-.0198<br>[-.0282, -.0114]<br>p<.001
Constant	.262<br>[.255, .268]<br>p<.001	.263<br>[.257, .270]<br>p<.001	.102<br>[.0890, .116]<br>p<.001	.0968<br>[.0836, .110]<br>p<.001	.371<br>[.365, .377]<br>p<.001	.236<br>[.230, .242]<br>p<.001	.357<br>[.350, .364]<br>p<.001	.500<br>[.494, .506]<br>p<.001
Standardization **of Math performance**	By country	Worldwide	By country	By country	By country	By country	By country	By country
Observations	251,120	251,120	301,360	301,360	301,360	301,360	256,841	289,536
R-squared	.0183	.0139	.0253	.0258	.0153	.0178	.0112	.0455

Notes: See notes of Table 1. 95% Confidence intervals in brackets. P-values based on two-sided t-tests.

Supplementary Table 9: Differential intentions to do math in France in 2016-2017: alternative measures of intentions and actual enrolment

	(1)	(2)	(3)	(4)	(5)	(6)	(7)
Sample :	Grades 10 and 12	Grade 10	Grade 10	Grade 12	Grade 12	Grade 12	Grade 12
Dependent variable:	Consider to do math, physics or computer sciences after high-school	Intends to go in a General scientific Grade 11 next year	Enrolled in a General scientific Grade 11 next year	Intends to go in a math-related education program after high-school	Enrolled in a math-related education program after high-school	Enrolled in a STEM program after high-school	Enrolled in a math-related selective program after high-school
Female	-.305<br>[-.318, -.291]<br>p<.001	-.102<br>[-.118, -.0865]<br>p<.001	-.0847<br>[-.0992, -.0702]<br>p<.001	-.297<br>[-.322, -.273]<br>p<.001	-.199<br>[-.221, -.177]<br>p<.001	-.168<br>[-.193, -.144]<br>p<.001	-.0679<br>[-.0824, -.0535]<br>p<.001
Math performance	.144<br>[.134, .153]<br>p<.001	.252<br>[.240, .264]<br>p<.001	.285<br>[.274, .295]<br>p<.001	.153<br>[.135, .170]<br>p<.001	.154<br>[.138, .169]<br>p<.001	.161<br>[.144, .178]<br>p<.001	.131<br>[.121, .141]<br>p<.001
**Female*(Math performance)**	-.0232<br>[-.0366, -.00975]<br>.001	-.00726<br>[-.0232, .00871]<br>.373	-.0393<br>[-.0538, -.0247]<br>p<.001	-.0668<br>[-.0915, -.0421]<br>p<.001	-.0777<br>[-.1000, -.0554]<br>p<.001	-.0757<br>[-.1000, -.0514]<br>p<.001	-.0638<br>[-.0783, -.0493]<br>p<.001
Constant	.523<br>[.513, .533]<br>p<.001	.543<br>[.532, .555]<br>p<.001	.446<br>[.436, .457]<br>p<.001	.420<br>[.402, .438]<br>p<.001	.385<br>[.369, .401]<br>p<.001	.472<br>[.455, .489]<br>p<.001	.130<br>[.120, .141]<br>p<.001
Observations	16,524	11,409	12,365	3,939	5,574	5,574	5,574
R-squared	.179	.256	.299	.203	.125	.103	.141

Columns (1) to (3) are based on a French general survey asking both 10th and 12th graders if they would consider pursuing math, physics or computer science after high school. It also analyzes the intentions of 10th graders to enroll in a scientific track in the following year after as well as their actual enrolment in such a track (in the following year, i.e., when they are in 11th grade). Columns (4) to (7) are based on a rich survey and on administrative data on 12th graders' educational choices. Standard errors in parentheses. 95% Confidence intervals in brackets. P-values based on two-sided t-tests.

Supplementary Table 10: Comparing sex gaps in math performance among adults in math-related, STEM-related jobs and in the whole adult population

a) Sex gap (female minus male) in the whole population			b) Sex gap among math or STEM workers			c) Ratio of Sex gaps (a/b)	d) Females-to-males ratio in top decile of whole population	e) Females-to-males ratio in top decile of math/STEM workers
a) math/STEM workers are individuals in math-related occupations								
**-0.226**	[-.247, -.204]	p<.001	**-0.263**	[-.348, -.177]	p<.001	1.164	0.618	0.255
b) math/STEM workers are individuals in STEM occupations								
**-0.226**	[-.247, -.204]	p<.001	**-0.210**	[-.281, -.138]	p<.001	0.928	0.618	0.248
c) math/STEM workers are individuals using math intensively at work								
**-0.226**	[-.247, -.204]	p<.001	**-0.274**	[-.331, -.217]	p<.001	1.212	0.618	0.275

Notes: The Table provides based on PIAAC for the adult population statistics similar to those provided in Table 2. See notes of Table 2 and Supplementary Figure 9.95% Confidence intervals in brackets. P-values based on two-sided t-tests.

Supplementary Table 11 : Differential intentions to do math of boys and girls (from Equation(1)). Investing the role of the comparative advantage in math versus reading

	(1)	(2)	(3)	(4)	(5)	(6)
	dependent variable is "strongly intending to do math"					
Girls	-.0345<br>[-.0432, -.0258]<br>p<.001	-.0257<br>[-.0342, -.0171]<br>p<.001	-.0257<br>[-.0343, -.0172]<br>p<.001	-.0267<br>[-.0356, -.0178]<br>p<.001	-.0390<br>[-.0522, -.0258]<br>p<.001	.0546<br>[-.162, .271]<br>p=.621
Math performance	.190<br>[.172, .209]<br>p<.001	.0409<br>[.0338, .0479]<br>p<.001	.0404<br>[.0332, .0477]<br>p<.001	.0376<br>[.0300, .0452]<br>p<.001	.0460<br>[.0387, .0532]<br>p<.001	.0417<br>[.0345, .0490]<br>p<.001
Girl*(Math performance)	-.0424<br>[-.0623, -.0225]<br>p<.001	-.0246<br>[-.0331, -.0161]<br>p<.001	-.0236<br>[-.0326, -.0145]<br>p<.001	-.0190<br>[-.0289, -.00911]<br>p<.001	-.0235<br>[-.0323, -.0148]<br>p<.001	-.0238<br>[-.0323, -.0152]<br>p<.001
Controls for ability :	Same as in Table 1, column 2	Comparative advantage (math minus reading)	Comparative advantage and its interaction with gender	Comparative advantage, its two-way interactions with math and gender, its three-way interaction with math and gender	A dummy for comparative advantage being positive and its interaction with gender	Quintiles of comparative advantage and their interactions with gender
Observations	251,120	251,120	251,120	251,120	251,120	251,120
R-squared	.0380	.0287	.0287	.0290	.0253	.0286

Notes: Analyses based on a sample of 61 countries. Math performance is standardized to have a weighted mean equal to 0 and a weighted standard deviation equal to 1 in each country of the sample (and on the full sample of countries). "Strongly intending to do math" corresponds to answering yes to five questions measuring PISA 2012 students' intentions to pursue a career in a math-related field or to invest more in math than in other science subjects or reading/literature. The rich set of controls in column 1 includes a third order polynomial in reading ability, a third order polynomial in science ability, reading ability interacted with gender, science ability interacted with gender, and interaction terms between math, science and reading ability (3 two-terms interactions and one three-term interaction). All estimates and standard errors are based on plausible values for math ability and account for measurement error in these abilities on top of standard sampling error. Standard errors in parentheses. 95% Confidence intervals in brackets. P-values based on two-sided t-tests.

Supplementary Table 12 : PIAAC (2015) participating countries and number of observations in each of them

Country	Number of observations	Percentage of whole sample
Austria	4,81	2.55
Belgium	5,122	2.72
Canada	25,016	13.28
Chile	4,887	2.59
Cyprus	4,738	2.51
Czech	5,721	3.04
Denmark	6,87	3.65
Estonia	7,155	3.80
Finland	5,123	2.72
France	6,556	3.48
Germany	5,465	2.90
Greece	4,618	2.45
Ireland	5,61	2.98
Israel	5,192	2.76
Italy	4,333	2.30
Japan	4,949	2.63
Korea	6,251	3.32
Lithuania	4,775	2.53
Netherlands	4,847	2.57
New Zealand	5,791	3.07
Norway	4,808	2.55
Poland	8,781	4.66
Russia	3,649	1.94
Singapore	5,127	2.72
Slovakia	5,366	2.85
Slovenia	4,998	2.65
Spain	5,677	3.01
Sweden	4,19	2.22
Turkey	4,948	2.63
UK	8,337	4.42
USA	4,697	2.49
Total	188,407	100.00
